# Ethyl Pyruvate Modulates Murine Dendritic Cell Activation and Survival Through Their Immunometabolism

**DOI:** 10.3389/fimmu.2019.00030

**Published:** 2019-01-28

**Authors:** Marita Chakhtoura, Robert W. Chain, Priscila Y. Sato, Connie C. Qiu, Michael H. Lee, Joseph J. Meissler, Toby K. Eisenstein, Walter J. Koch, Roberto Caricchio, Stefania Gallucci

**Affiliations:** ^1^Laboratory of Dendritic Cell Biology, Department of Microbiology-Immunology, Lewis Katz School of Medicine, Temple University, Philadelphia, PA, United States; ^2^Center for Translational Medicine, Lewis Katz School of Medicine, Temple University, Philadelphia, PA, United States; ^3^Department of Pharmacology, Lewis Katz School of Medicine, Temple University, Philadelphia, PA, United States; ^4^Department of Microbiology-Immunology, Lewis Katz School of Medicine, Temple University, Philadelphia, PA, United States; ^5^Center for Substance Abuse Research, Lewis Katz School of Medicine, Temple University, Philadelphia, PA, United States; ^6^Division of Rheumatology, Department of Medicine, Lewis Katz School of Medicine, Temple University, Philadelphia, PA, United States

**Keywords:** dendritic cells, ethyl pyruvate, TLR, DC activation, metabolism

## Abstract

Attenuating the innate immunity activation could ameliorate inflammation and disease in settings such as transplant rejection or autoimmunity. Recently, a pivotal role for metabolic re-programming in TLR-induced dendritic cell (DC) activation has emerged. Ethyl pyruvate (EP), a pyruvate derivative, possesses anti-inflammatory properties *in vitro* and in animal models of disease. However, its effects on DCs remain elusive. We found that EP attenuated LPS-induced activation of murine GM-CSF bone marrow-derived dendritic cells (DCs) *in vitro*, reducing pro-inflammatory cytokine and IL-10 production, costimulatory molecule and MHC expression, the type I Interferon (IFN-I) response, the LPS-induced cell death, and the ability of DCs to stimulate allogeneic T cells. DC activation induced by TLR7 and TLR9 ligands was also suppressed by EP *in vitro*. Finally, EP decreased TLR-induced activation stimulated *in vivo* in conventional DCs and inflammatory monocytes. Investigating EP mechanisms, we found that EP decreased glycolysis and mitochondrial respiration, upon and in absence of TLR stimulation, by reducing ERK, AKT, and nitric oxide (NO) activation. These results indicate that EP inhibits most of the DC biological responses to TLR triggering, altering the metabolic reprogramming necessary for DC activation.

## Introduction

GM-CSF-dendritic cells (which we will refer to as DCs in this paper) are an experimental model of inflammatory DCs (1), capable of sensing pathogen-associated molecular patterns (PAMPs) and damage-associated molecular patterns (DAMPs), hence initiating the activation/maturation process (2–5). During activation, DCs up-regulate costimulatory molecules and secrete cytokines in order to present major histocompatibility complex (MHC)-restricted antigens to T lymphocytes in a pro-inflammatory context and induce an immune response. Pathological conditions, such as graft rejection, autoimmune diseases, and excessive inflammation warrant the dampening of dendritic cell activation to limit damaging immune responses.

Lipopolysaccharide (LPS), a component of the outer membrane of Gram-negative bacteria, is a prototypic PAMP, a toll-like receptor 4 (TLR4) ligand and a potent DC activator. LPS stimulation has been shown to induce a metabolic reprogramming, which sustains GM-CSF-DC activation (6, 7). These DCs in a resting state support their energy needs through fatty acid oxidation to fuel the oxidative phosphorylation (OXPHOS) occurring in the mitochondria (7). Although these cells consume glucose, it is unclear if it is primarily for OXPHOS or for anabolism (8). Pearce et al. have reported that DCs, as well as macrophages (9), undergo a rapid switch to aerobic glycolysis soon after TLR engagement, with a sustained mitochondrial respiration observed during the first 30 min of stimulation, which progressively decreases after 8–12 h. The up-regulation of glycolysis in the early DC activation phase is essential for NADPH regeneration and fatty acid synthesis (7, 8). Lipogenesis, indispensable for DC activation, enables the enlargement of the endoplasmic reticulum and Golgi to support protein synthesis and shuttle the newly synthesized cytokines and inflammatory mediators. This phase of activation is TBK1/IKKε/AKT-mediated, whereby AKT directly phosphorylates the key glycolytic enzyme hexokinase II (HK-II), to induce its activity (7, 10). As for the later activation phase, glycolysis up-regulation is maintained in iNOS-expressing DCs secondary to the autocrine production of nitric oxide (NO), which can suppress mitochondrial respiration (6, 11). Hence, around 24 h post-stimulation, glycolysis sustains DC survival, and the cells continue to function as Ag-Presenting Cells (12).

Ethyl pyruvate (EP) is an aliphatic ester derivative of pyruvate with a higher ability to enter cells and access mitochondria (13, 14). It was first proposed by Sims et al. to improve mesenteric ischemia/reperfusion in rats (15). With its ROS-scavenging properties (16–20), EP has been shown to possess anti-inflammatory properties in various cell types *in vitro* (21–25) and in many animal models of disease, such as severe sepsis, ischemia reperfusion injury, hemorrhagic shock, stroke, and others (15, 19, 21, 22, 24, 26–28). EP can decrease the production of pro-inflammatory cytokines by targeting different signaling pathways, the most important of which is the NF-kB pathway (13, 17, 29, 30). In addition, EP was reported to be a relatively safe agent at clinically relevant doses when evaluated in a study of endotoxemic vs. normal horses (31), as well as in a clinical trial of patients with cardiopulmonary bypass (32). Based on its similarities with pyruvate and methyl pyruvate, EP may act as the first substrate of the citric acid cycle, also known as TCA or Krebs cycle, and by extension drive mitochondrial respiration (13). To date, the effect of EP on DC responses, as well as the link between EP and immunometabolism, remain unknown.

Here we show for the first time that EP inhibits the activation of murine DCs, generated in culture in the presence of GM-CSF, considered a model of inflammatory DCs (1). We found that EP suppresses TLR-induced cytokine production, up-regulation of costimulatory molecules, as well as the IFN-I response. We show that EP decreases DC immunometabolism by inhibiting the LPS-induced switch to glycolysis and decreasing mitochondrial respiration as well, without reducing DC survival. This decreased metabolism is mediated by the reduction of AKT and ERK1/2 phosphorylation, normally induced by TLR stimulation in the early DC activation phase. Moreover, EP also affects the late DC activation phase by suppressing their production of NO. Furthermore, we show that EP reduces DC ability to stimulate allogeneic T cells *in vitro* and to respond to TLR stimulation *in vivo*. Therefore, EP is a novel suppressor of DC activation with the therapeutic potential of immunosuppression by blocking DC metabolic reprogramming.

## Materials and Methods

### Mice

Female C57BL/6 (B6) and C3HeB/FeJ mice were purchased from the Jackson Laboratory (Bar Harbor, ME, USA) and maintained in our colony in accordance with the guidelines of the Institutional Animal Care and Use Committees of our University, a member of the American Association for the Accreditation of Laboratory Animal Care-accredited facilities. Mice were used between 6 and 12 weeks of age.

### *In vitro* Bone Marrow-Derived DC Cultures

Bone marrow precursor cells were flushed from femurs and tibias of B6 mice and differentiated into DCs in presence of GM-CSF as described in the Supplemental Procedures (33, 34). The differentiated DCs were stimulated on day 6 or 7 with ethyl pyruvate (EP) (Sigma-Aldrich) and/or the TLR ligands LPS (100 ng/ml), R848 (1 μg/ml) or CpG B (10 μg/ml) 1 h later. In select experiments, EP treatment was delayed and followed LPS stimulation by 1 h.

### Assessment of Dendritic Cell Viability and Activation by Flow Cytometry

DCs were analyzed by flow cytometry for cell viability and the expression of surface costimulatory markers as well as MHC molecules. Briefly, cells were stained with Annexin V in Annexin V-binding buffer for 15 min before the addition of 7-AAD. Alternatively, cells were incubated with Fc**γ**R blocker (purified anti-mouse CD16/CD32, clone 93) for 15 min and then stained with fluorochrome-conjugated antibodies against DC surface markers for 30 min on ice. The antibodies used were directed against mouse CD11c (N418), CD86 (GL-1), CD11b (M1/70), CD40 (HM40-3), CD80 (16-10A1), MHC-I (H2kb) (28-8-6), and MHC-II (M5/114.15.2). Cells were fixed in 2% paraformaldehyde in PBS and analyzed on a FACSCanto flow cytometer (BD Bioscience) with FlowJo software (Tree Star, Ashland, OR, USA). In experiments where EP was added after LPS, flow cytometry was performed 24 h after EP treatment.

### Measurement of Cytokine Levels by ELISA

Supernatants were collected from DC cultures post-TLR stimulation or EP treatment for the measurement of IL-12p70, TNF-α, IL-6, and IL-10 levels using the BD Pharmingen ELISA kits and CXCL-10 levels using the R&D kit, according to the manufacturer's protocol (see Supplemental Procedures). Optical densities were measured at 450 nm and results analyzed with SoftMax Pro software (Molecular Devices Corporation, Sunnyvale, CA).

### Gene Expression Quantification by qRT-PCR

Gene expression of *in vitro* DCs was analyzed by quantitative reverse transcription PCR (qRT-PCR) using Taqman probes. Total RNA was extracted from DCs harvested 1 and 6 h after TLR stimulation using the Quick-RNA MiniPrep kit (Zymo Research) and then was reverse transcribed using the High Capacity cDNA RT kit. Pre-made Taqman primers and probes (Applied Biosystems) were used to assess expression of *Ifnb, Mx1, Isg15, Irf7, Cxcl10*, and *Inos*. Cyclophillin (*Cyc*) was used as the housekeeping gene for normalization. The Ct method of relative quantification of gene expression was used for analysis and the normalized Ct values (against cyclophillin) were calibrated against the control sample (untreated DCs) in each experiment. In experiments where EP was used post-LPS stimulation, qRT-PCR analysis was performed 1 and 6 h after EP treatment.

### Western Blot Analysis

Thirty micrograms of denatured total lysate were used in SDS-PAGE before transfer to PVDF membranes. Membranes were incubated overnight at 4°C with primary antibodies against the following phosphorylated and total proteins: ERK 1/2, p38, JNK 1/2, and AKT, as well as total IKBa and IKBb. Rabbit and mouse anti-mouse actin, and mouse anti-mouse GAPDH were used as loading controls. Membranes were then incubated with IR Dye 800 goat anti-rabbit and IR Dye 680 donkey anti-mouse (LI-COR Biosciences) diluted in blocking buffer plus 0.1% Tween-20. Proteins were visualized by scanning the membranes on an Odyssey Infrared Imaging System (LI-COR Biosciences) in both 700 and 800 nm channels.

### Mixed Lymphocyte Reaction Assay

DCs from B6 female mice were generated in culture as described above and treated on day 6 with or without 10 mM EP and/or 100 ng/ml LPS 1 h later. After 24 h, DCs were washed and added to allogeneic APC-depleted C3HeB/FeJ female splenocytes (800,000 cells per well) in a DC:splenocyte ratio of 1:30. Each condition of DC:splenocyte was run in quadruplicate. B6 DCs alone from each condition and C3HeB/FeJ splenocytes alone served as controls. After 48 h, cells were pulsed with 1 μCi of H^3^ thymidine for 18 h, harvested and counted with a Tri Carb liquid scintillation counter (PerkinElmer, Waltham, MA) for radioactivity in counts per minute (cpm). Cpm recorded for every condition was subtracted from that of the appropriate control DCs. Stimulation Index was calculated using the formula cpm of a sample/cpm of splenocytes incubated with DCs left untreated in the absence of EP and LPS.

### Metabolism Assays

Real time analysis of oxygen consumption rate (OCR) and extracellular acidification rate (ECAR) was performed using the Seahorse XF-96 metabolic extracellular flux analyzer (see Supplemental Procedures). Metabolism assays were run and ECAR and OCR measurements were recorded at an acute (30 min) and 24 h time points. For the acute time point, plots were generated based on the values recorded at rate 7 of the assay, which is equivalent to 30 min after addition of LPS or medium for the cells that did not receive LPS. This also coincides with 30 min after the beginning of the Seahorse run. As for the 24 h time point, plots were generated based on the values recorded 24 h following the addition of LPS or medium for the cells that did not receive LPS. This time point is equivalent to the rate 7 of the assay, coinciding with 30 min after the beginning of the run. For the 24 h time point, DCs were generated as above and either left untreated, treated with EP (10 mM) and/or LPS (100 ng/ml) for 24 h. The cells were then re-plated at a density of 70,000 cells per 70 μl per well of XF-96 culture plates previously coated with poly-d-lysine for adherence. Cells were then washed in XF assay medium and analyzed for OCR and ECAR in response to 1 μM oligomycin, 1.5 μM fluoro-carbonyl cyanide phenylhydrazone (FCCP), and 100 nM rotenone plus 1 μM antimycin A as part of the Seahorse XF Cell Mito Stress Test kit (Agilent, Santa Clara, CA). The use of the Mito Stress Test Kit is an acceptable method for ECAR measurement (35–37) since it is able to measure the acidification caused by the H^+^ ions in the milieu. For the acute time point, DCs were harvested and treated directly in the XF-96 culture plates.

### Nitric Oxide Quantification

Nitrite concentration was measured as a proxy for nitric oxide (NO) in DC culture supernatants. A colorimetric assay using the Griess reagent (Acros Organics) was utilized to measure nitrite levels in uM according to the manufacturer's protocol. Absorbance was measured at 550 nm and a standard curve was generated.

### *In vivo* EP Injection and Spleen and Lymph Node Cell Staining

C57BL/6 mice were injected i.p. with 80 mg/kg of EP in 200 μl PBS (vehicle) 1 h before the injection of 30 μg/mouse of TLR7 ligand R848 in 200 μl PBS (vehicle). EP was further administered 4, 8, and 20 h after R848 stimulation. Spleens and mesenteric lymph nodes (mLN) were harvested 24 h post-R848 stimulation and single cell suspensions were prepared using DNase I (Sigma-Aldrich) and collagenase IV (Worthington Biochemical Corporation) as well as ACK red blood cell lysis buffer for spleens (VWR). Cell viability was assessed by either staining with fixable viability dye eFluor780 (eBioscience) or 7-AAD. Cells were incubated with purified rat anti-mouse CD16/CD32 monoclonal antibody (clone 2.4G2) prior to the addition of fluorochrome-conjugated antibodies against conventional DC/inflammatory monocyte surface markers: rat anti-mouse CD11c (N418), CD86 (GL-1), Ly-6C (HK1.4), and CD11b (M1/70). Cells not stained with 7-AAD were fixed with 1% paraformaldehyde. Samples were acquired on a FACSCanto Cytometer then analyzed with FlowJo.

### Statistical Analyses

Prism 6 (GraphPad software, San Diego, CA, USA) was used for data analysis. Means and Standard Errors (Mean ± SEM) were calculated by averaging results from independent experiments. Throughout the manuscript, “n” refers to independent cell cultures from individual mice, and each result from each culture is the mean of two-three biological replicates. Statistical significance was determined using unpaired two-tailed Student's *t*-test or ratio paired two-tailed Student's *t*-test for comparison between two groups. One-way ANOVA and Two-way ANOVA were used for multiple comparisons followed by the Bonferroni and Sidak multiple comparisons *post-hoc* correction tests, respectively. *P*-values of *p* < 0.05 (marked in the figures as ^*^P < 0.05, ^**^P < 0.01, ^***^P < 0.001, and ^****^P < 0.0001) were considered significant.

## Results

### Ethyl Pyruvate Suppresses the Activation of GM-CSF-Dendritic Cells (DCs)

**Figure 1:**
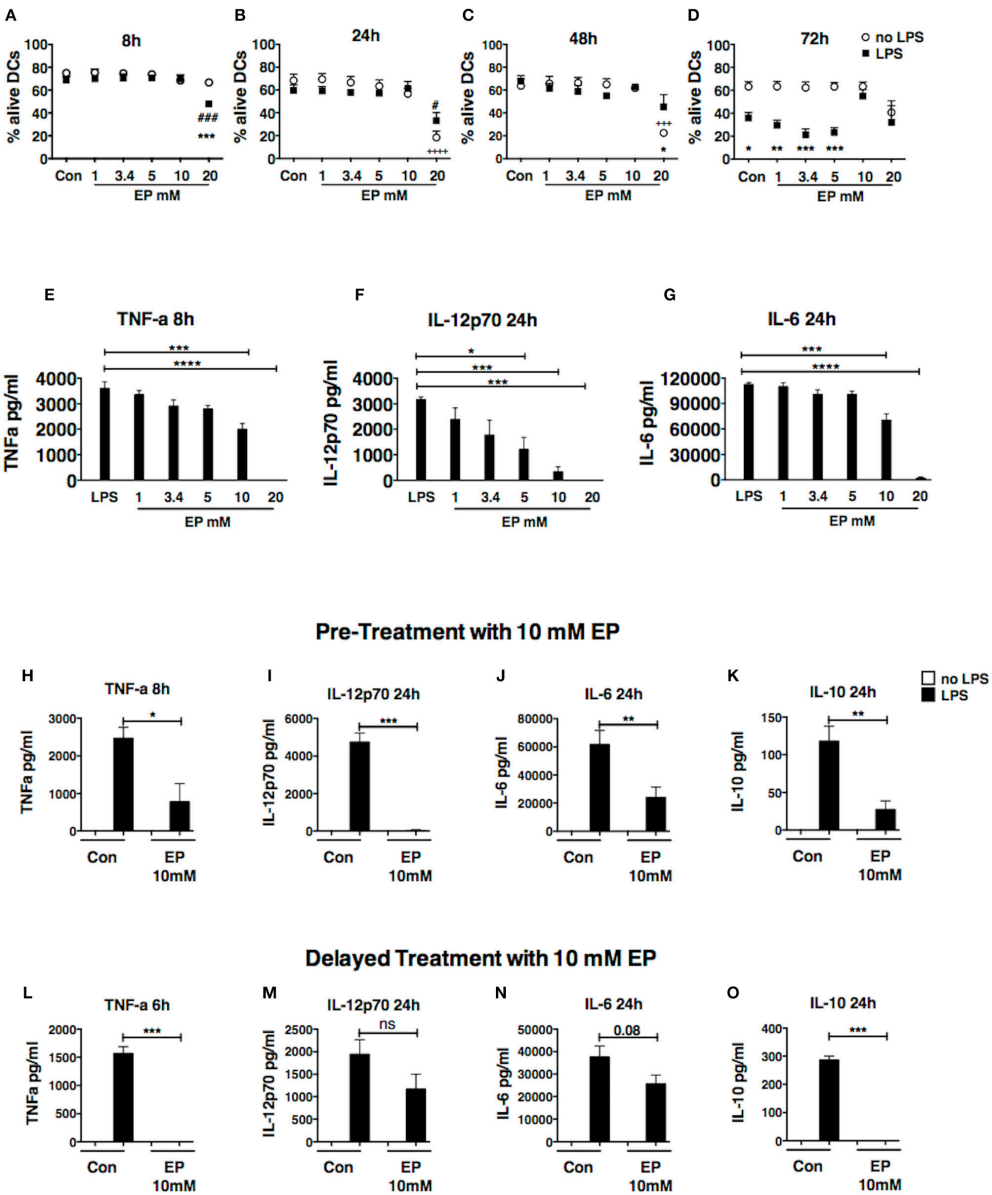
Ethyl pyruvate (EP) dose titration response. Dose titration response to EP in DCs in presence or absence of 100 ng/ml LPS. EP was administered 1 h prior to LPS stimulation **(A–D)** DC viability. Cells were stained with AnnexinV and 7-AAD to assess viability by flow cytometry. Results are shown as percent Annexin V-7AAD double negative cells (alive) in the CD11c gate. Statistical significance was analyzed using One-way ANOVA followed by the Bonferroni multiple comparisons test and is shown by the symbols + between conditions in absence of LPS and the untreated control, and by the symbol # between conditions in presence of LPS and the LPS control. Statistical significance was also analyzed using Two-way ANOVA followed by the Sidak multiple comparisons test and is shown by the symbol * between conditions with and without LPS in presence of the same EP concentration **(E–G)** Cytokine production. Culture supernatants were analyzed by ELISA for levels of TNF-a at 8 h as well as IL-12p70 and IL-6 at 24 h post-LPS stimulation. Results are the average of 2 independent experiments with two biological replicates per experiment (*n* = 2, 4 data numbers). Data are shown as mean ± SEM. Statistical significance was analyzed using One-way ANOVA followed by the Bonferroni multiple comparisons test. **(H–O)** DCs were treated with 10mM EP either 1 h before **(H–K)** or 1 h after **(L–O)** LPS stimulation, and supernatants were analyzed for **(H,L)** TNF-a, **(I,M)** IL-12p70, **(J,N)** IL-6, **(K,O)** IL-10 by ELISA. Results are shown as mean ± SEM from 4 to 5 independent experiments (*n* = 4–5) **(H–K)** and 3 independent experiments (*n* = 3) **(L–O)**. Data were analyzed using the two-tailed unpaired Student's *t*-test. *P*-values *P* < 0.05 were considered of statistical significance. *P < 0.05, **P < 0.01, ***P < 0.001, and ****P < 0.0001.

To determine the effect of EP on DC survival and activation, we treated bone marrow-derived DCs, generated in culture in GM-CSF-enriched medium, with a dose titration of EP and we stimulated them 1 h later with 100 ng/ml of LPS. We harvested the DCs 8, 24, 48, and 72 h after LPS stimulation. We first evaluated DC viability by flow cytometric analysis of the staining with AnnexinV and 7-AAD and found that the dose of 10 mM EP did not affect DC viability at 8, 24, or 48 h (Figures 1A–C) and resulted in the strong inhibition of pro-inflammatory cytokine production, which was significant when compared to LPS alone (Figures 1E–G). We measured the prototypic Th1 cytokine IL-12p70 as well as the pro-inflammatory IL-6 at 24 h, and TNF-α at 8 h. We observed similar findings with TNF-α at 24 h, as well as IL-12p70 and IL-6 at 48 and 72 h (Supplemental Figures 1A–C). Furthermore, LPS is known to induce DC death 72 h post-stimulation (38) and EP 10 mM was able to rescue DCs from LPS-induced cell death (Figure 1D). The lower doses of EP, namely 1, 3.4, and 5 mM, did not induce cell death in the presence or absence of LPS at the different time points tested, and were also unable to rescue the cells from the 72 h LPS-induced death (Figures 1A–D). When measuring cytokine levels, we found a dose-dependent decrease in IL-12p70 at 24 h (Figure 1F), but also at 8, 48, and 72 h (data not shown), starting with the lowest EP dose (1 mM) and reaching significance with the 3 highest doses. The 3.4 and 5 mM doses caused a modest reduction in TNF-α levels at the 8 h (Figure 1E) and 24 h time points (data not shown), and IL-6 at all time points (Figure 1G and data not shown). On the other hand, the highest tested dose of EP (20 mM) killed DCs as early as 8 h post-LPS stimulation (Figures 1A–D) and no cytokine production was detected at any time point (Figures 1E–G). Altogether, these data suggest that 10 mM is the optimal EP dose to test on DC responses, hence this dose was used in the rest of our experiments.

### Pre- and Delayed Treatments With Ethyl Pyruvate Inhibit Cytokine Production in LPS-Stimulated DCs

In order to explore whether EP affects early events downstream of TLR, we then compared the effect of EP as pre-treatment, administered 1 h before LPS stimulation, and as delayed treatment, administered 1 h after LPS stimulation. As pre-treatment, EP 10 mM completely abrogated the production of IL-12p70 at 24, 48, and 72 h (Figure 1I and Supplemental Figure 1A), and induced a significant reduction in the production of pro-inflammatory TNF-α and IL-6 (Figures 1H,J and Supplemental Figures 1B,C). We also found that EP inhibited the production of the anti-inflammatory cytokine IL-10 at all studied time points (Figure 1K and Supplemental Figure 1D). These results suggest that EP can suppress DC cytokine production in general and at doses that do not compromise cell viability. Similar to the other cytokines, no IL-10 was detected in presence of 20 mM EP, when many of the DCs were dead (data not shown).

To determine whether pre-treatment with EP was necessary for its ability to inhibit DC activation, we assessed the effect of delayed treatment with EP, administrating it 1 h after LPS stimulation, and found that it was likewise very potent in reducing DC activation. The total abrogation of TNF-α at 6h (Figure 1L) and strong inhibition at 24 h (Supplemental Figure 1E), as well as IL-10 suppression (24 h; Figure 1O), show the potent effect on cytokine production even after LPS had started signaling DC activation. We also observed a smaller but not significant inhibition of IL-12p70 and IL-6 (24h; Figures 1M,N). These findings suggest the possibility that EP (10 mM) inhibits different cytokines through different mechanisms, modifying different molecular targets.

### Ethyl Pyruvate Decreases DC Activation Upon LPS-Stimulation by Targeting Costimulation

**Figure 2:**
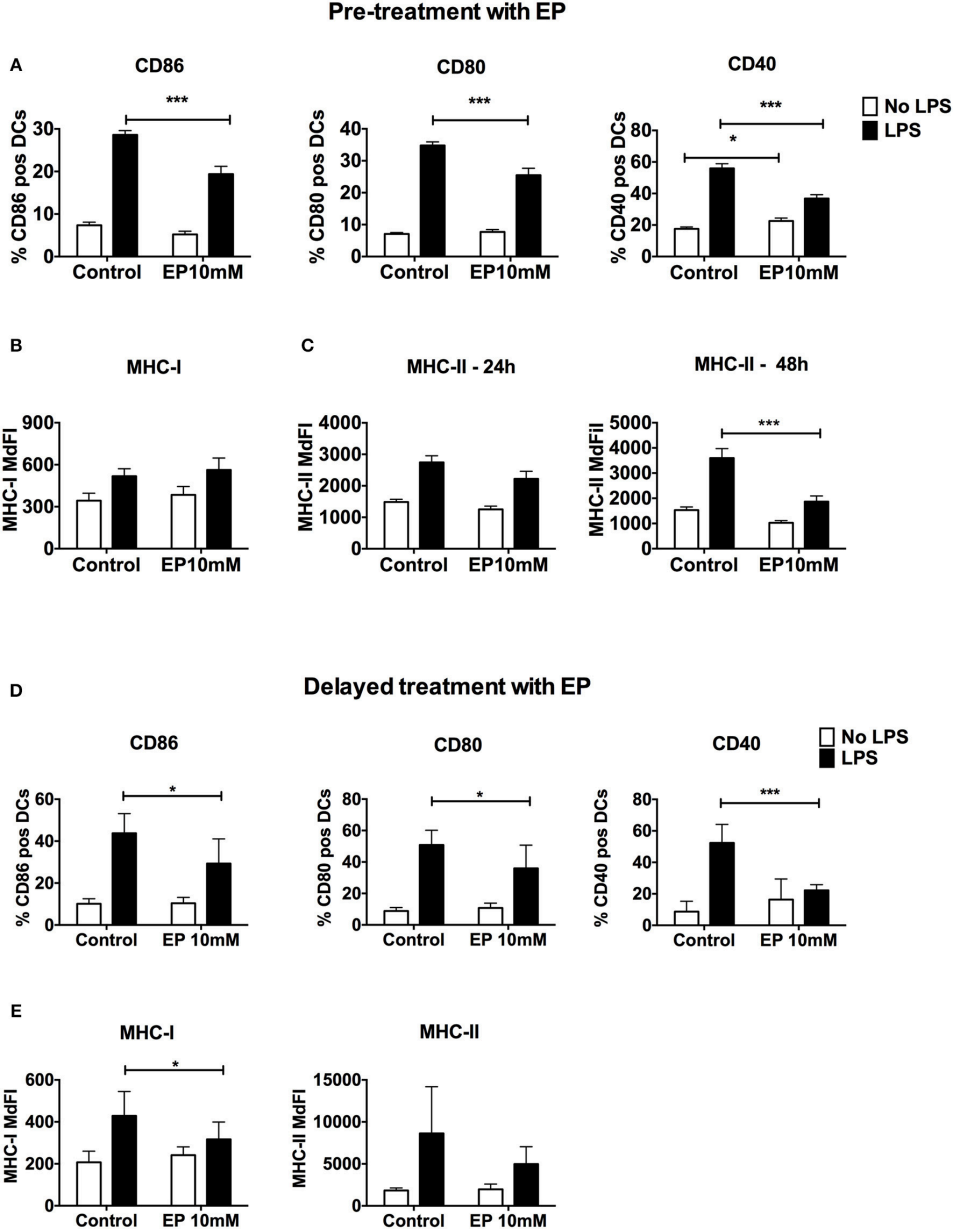
EP decreases LPS-induced DC up-regulation of surface costimulatory molecule and MHC expression. **(A–C)** Pre-treatment with EP. DCs were pre-treated with 10mM EP for 1 h before stimulation with LPS 100 ng/ml, then were harvested 24 h **(A–C)** and 48 h **(C)** later and analyzed by flow cytometry for surface costimulatory molecule CD86, CD80, CD40, and MHC class I and Class II expression. Results are expressed as mean ± SEM of at least 6 independent experiments (*n* = 6). **(D,E)** Delayed treatment with EP. DCs were stimulated with 100 ng/ml LPS followed by 10mM EP 1 h later, then analyzed after 24 h by flow cytometry. Results are expressed as mean ± SEM of 4 independent experiments (*n* = 4). Data were analyzed using the two-tailed unpaired Student's *t*-test. *P*-values *P* < 0.05 were considered of statistical significance. *P < 0.05, **P < 0.01, ***P < 0.001, and ****P < 0.0001.

Since the up-regulation of costimulatory molecules and MHC expression is fundamental for DC activation and antigen presentation, we assessed cell surface expression of these molecules upon treatment with EP by flow cytometry. EP administered before TLR triggering caused a significant decrease in LPS-induced up-regulation of CD86, CD80, and CD40 expression at the 24 h time point (Figure 2A). We also detected a significant increase in CD40 expression with EP alone when compared with the unstimulated control (Figure 2A). Moreover, we observed a delay in the inhibition of MHC-II surface expression by EP, which was significant at 48 h but not at 24 h post-LPS stimulation (Figure 2C). EP did not decrease MHC-I expression at any time point (Figure 2B and data not shown). Furthermore, the delayed EP treatment induced a significant decrease in costimulatory molecule expression (CD86, CD80, and CD40) 24 h post-EP (Figure 2D) as well as a significant reduction in MHC-I but not MHC-II expression (Figure 2E and data not shown).

These results indicate that EP suppresses DC activation not only by decreasing Signal 3, namely cytokine production, but also by reducing the ability of the DCs to provide Signal 2, and to a lesser extent Signal 1, the expression of costimulatory molecules and MHC, respectively. These results may have important consequences on the ability of DCs to present antigens and stimulate T cell responses.

### Ethyl Pyruvate Decreases the IFN-I Response in LPS-Activated DCs

**Figure 3:**
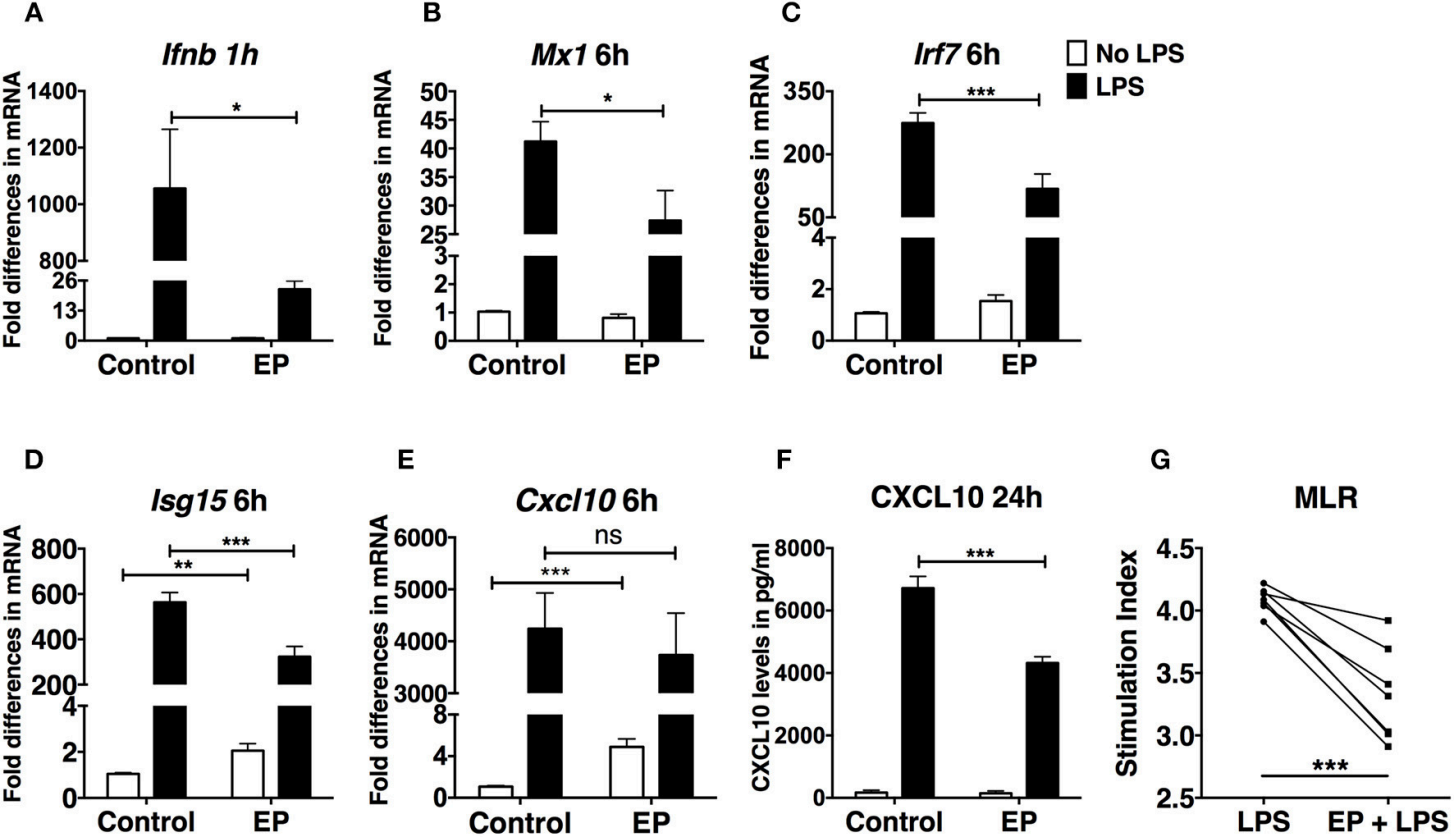
EP decreases the IFN-I response in LPS-stimulated DCs. DCs were pre-treated with 10mM EP for 1 h before LPS 100 ng/ml. Cells were harvested after **(A)** 1 h for *Ifnb* level of expression and **(B–E)** 6 h for the IFN-I-stimulated gene expression analysis by real-time RT-PCR. Values were expressed as fold difference in mRNA from the control (untreated cells). **(F)** CXCL10 ELISA from 24 h culture supernatants. Results in **(A)** are the average of biological replicates from 1 representative of 5 independent experiments (*n* = 5). Results in **(B–E)** are from 10 independent experiments (*n* = 10) and in **(F)** from 5 independent experiments (*n* = 5). Results are expressed as mean ± SEM. **(G)** EP reduces the ability of DCs to stimulate an allogeneic T cell response in the MLR assay. We treated DCs with LPS ± EP pre-treatment for 24 h, then we washed and used them as APCs to induce lymphocyte proliferation in an *in vitro* MLR assay. We used a DC:splenocyte ratio of 1:30. Lymphocyte proliferation was measured by ^3^H thymidine incorporation. Stimulation index was calculated by dividing the counts per minute (cpm) for each condition by the cpm of lymphocytes exposed to untreated DCs. Results are from 3 to 4 biological replicates of 2 independent experiments (*n* = 2, 7 data numbers). Data was analyzed using the two-tailed unpaired Student's *t*-test. *P < 0.05, **P < 0.01, ***P < 0.001, and ****P < 0.0001.

Type I IFNs are crucial for DC activation and antigen presentation (39, 40). DCs respond to and produce IFN-I, which act in an autocrine fashion to enhance DC activation (41). IFN-I lead to the activation of interferon-stimulated genes (ISGs), which boost DC activation and their stimulation of T cells. Therefore, we performed real time RT-PCR on DCs pre-treated with EP followed by LPS 1 h later to assess gene expression of *Ifnb* and ISGs. EP drastically decreased *Ifnb* expression in LPS-activated DCs (Figure 3A). The same trend was consistently observed in 5 independent experiments. However, we show a representative plot due to commonly observed variation in *Ifnb* expression between experiments. We then measured the expression of ISGs 6 h after LPS stimulation and found that EP significantly reduced *Mx1, Irf7, and Isg15* expression (Figures 3B–D). Surprisingly, EP could not decrease the LPS-induced transcript up-regulation of the IFN-I-dependent chemokine *Cxcl10* and when administered alone to the cells, EP significantly increased *Cxcl10* expression as compared to control (Figure 3E). The latter observation was also detected with *Isg15* (Figure 3D). However, when we measured the 24 h CXCL10 protein levels by ELISA, we found that EP significantly decreased its LPS-induced production (Figure 3F). We found comparable inhibition of CXCL10 production, as well as of *Ifnb* and ISG transcript levels upon delayed EP treatment (data not shown). These results indicate that EP decreases the IFN-I response in LPS-stimulated DCs, further attenuating their activation. These results suggest that EP affects DC responses more at the level of protein translation/secretion for some key molecules. This conclusion is valid even if EP was administered in a delayed fashion.

### Ethyl Pyruvate Reduces DC Ability to Stimulate an Allogeneic T Cell Response *in vitro*

To understand the functional consequences of the suppressive effects of EP on the capacity of DCs to function as Ag-Presenting Cells (APCs), we studied their ability to stimulate an allogeneic T cell response in an *in vitro* Mixed Lymphocyte Reaction (MLR) assay. APC-depleted splenic lymphocytes from female C3HeB/FeJ mice (haplotype H-2k^k^) were incubated with murine female C57BL/6 DCs (haplotype H-2k^b^), which were either untreated or pre-treated for 1 h with EP and/or LPS, 24 h before use in the MLR. We found, as expected, that LPS-treated DCs were efficient in inducing lymphocyte proliferation with a high Stimulation Index (SI), which was significantly decreased with EP (Figure 3G).

These results indicate that EP decreases DC ability to function as APCs, supporting a functional consequence for the decreased up-regulation of costimulatory molecules and cytokine production presented in Figures 1, 2.

### Ethyl Pyruvate Inhibits the Activation of DCs Stimulated by Other TLR Ligands

**Figure 4:**
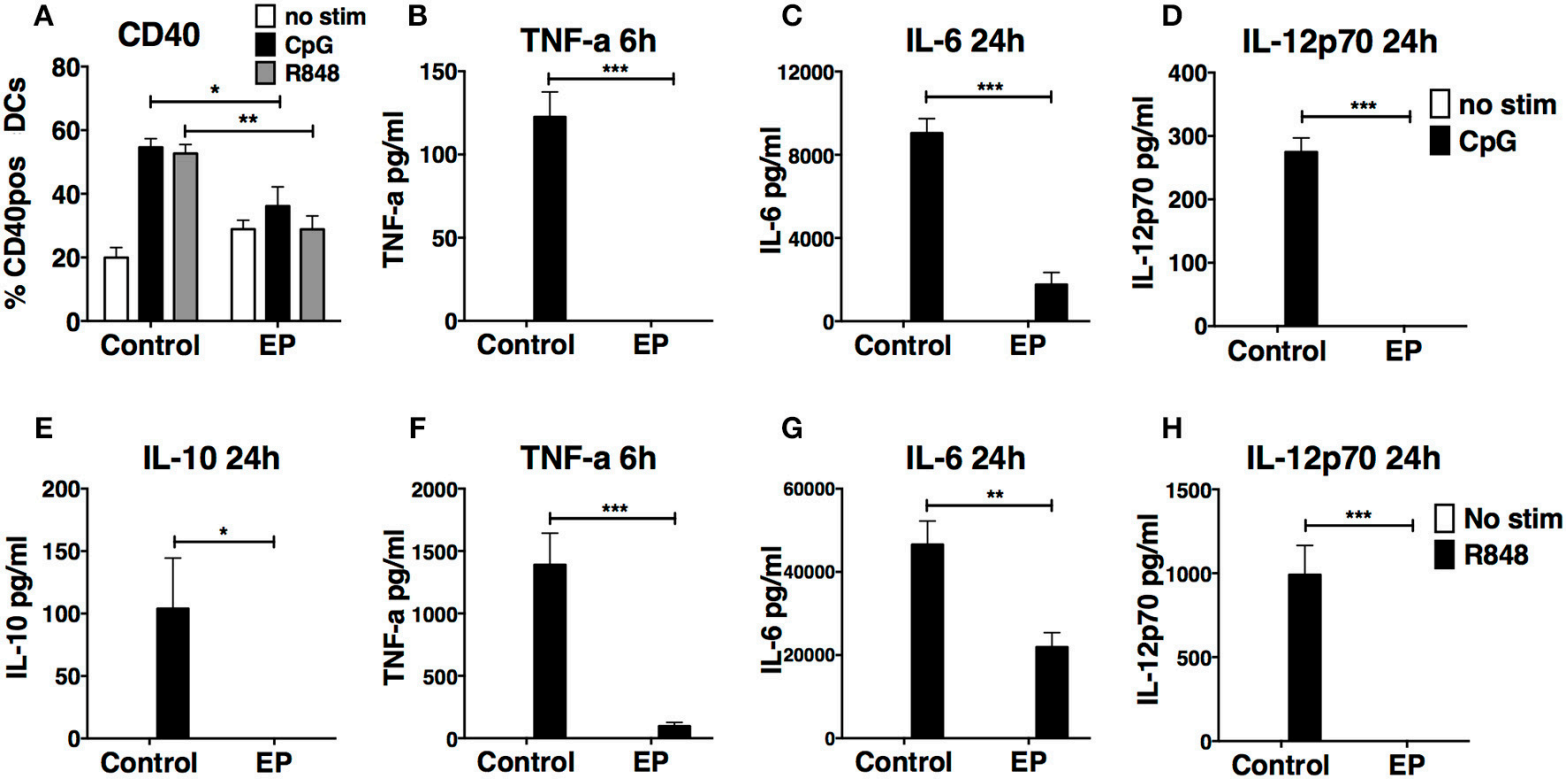
EP suppresses CpG B- and R848-induced DC activation by decreasing cytokine production and surface costimulatory molecules. DCs were pre-treated with 10mM EP for 1 h before stimulation with CpG B 10 μg/ml or R848 1 μg/ml. Cells and supernatants were harvested 24 h and 6 or 24 h post-stimulation, respectively, and analyzed by ELISA for cytokine levels and by flow cytometry for surface costimulatory molecule expression. EP decreases CD40 expression **(A)** and production of TNF-a **(B,F)**, IL-6 **(C,G)**, IL-10 **(E)**, and IL-12p70 **(D,H)** upon CpG B **(A–D)** and R848 **(A,E–H)** stimulation. Results are the average of biological replicates from 2 to 3 independent experiments (*n* = 2–3) and are expressed as mean ± SEM. Data was analyzed using the two-tailed unpaired Student's *t*-test. *P < 0.05, **P < 0.01, ***P < 0.001, and ****P < 0.0001.

We next tested the effect of 1 h pre-treatment with EP on the DC response to the stimulation by two other TLRs, TLR9, and TLR7, using CpG B and R848, respectively. We found that EP inhibited the production of TNF-α, IL-6, and IL-12p70 upon CpG B (Figures 4B–D) and R848 (Figures 4F–H) stimulation. Moreover, EP completely suppressed the production of the anti-inflammatory cytokine IL-10 induced by R848 stimulation (Figure 4E). Pre-treatment with EP also affected the up-regulation of costimulatory molecules upon DC activation with TLR9 and TLR7 ligands, as shown by the significant decrease in CD40 up-regulation induced by CpG B and R848 stimulation (Figure 4A). These findings show that the effect of EP is not specific to LPS stimulation and that it inhibits the general activation induced by TLR triggering.

### Ethyl Pyruvate Does Not Affect IKB Degradation but Targets ERK Phosphorylation

**Figure 5:**
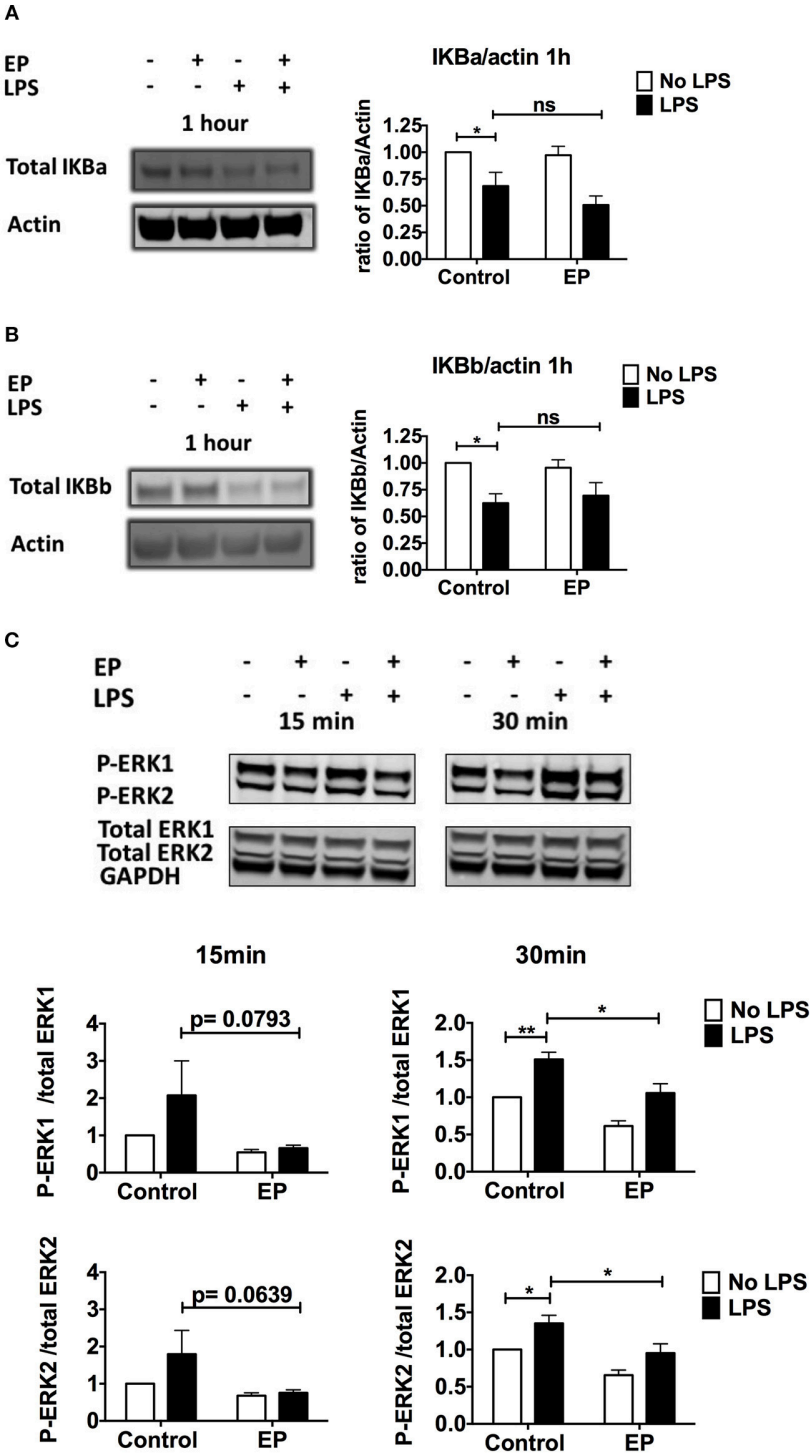
EP does not affect IKB degradation but decreases ERK phosphorylation in LPS-stimulated DCs. DCs were pre-treated with 10mM EP for 1 h before 100 ng/ml LPS. **(A,B)** Cells were harvested 1 h after LPS stimulation and IKBa **(A)** and IKBb **(B)** protein expression was detected by western blotting. Densitometry was analyzed by calculating the ratio of IKBa or IKBb to actin. Results are normalized to the untreated control and shown as mean ± SEM of 5–6 independent experiments (*n* = 5–6). **(C)** p-ERK/Total ERK detection. Cells were harvested 15 and 30min after LPS stimulation and ERK1/2 protein expression was detected by western blotting. Densitometry was analyzed by calculating the ratio of phosphorylated p-ERK to total ERK. GAPDH was included as a confirmation control only. Results are normalized to the untreated control and shown as mean ± SEM of 7 independent experiments (*n* = 7). Data was analyzed using the ratio paired two-tailed Student's *t*-test. *P < 0.05, **P < 0.01, ***P < 0.001, and ****P < 0.0001.

To determine whether EP affects the signaling pathway downstream of TLR activation in DCs, we first studied the activation of the NF-kB pathway, one of the three major signaling pathways stimulated by TLR triggering. We performed western blots on lysates of DCs pre-treated with EP and LPS and harvested 1 h post-LPS stimulation. We measured IKBa and IKBb degradation as a proxy for NF-kB activation and we found, in agreement with the literature, that 1 h of LPS stimulation decreased IKBa and IKBb total levels relative to unstimulated DCs (Figures 5A,B). EP did not change this LPS-induced decrease in IKBa/IKBb protein levels, shown in Figures 5A,B as representative blots (left) and as mean of 5–6 independent experiments (right).

A second important signaling pathway downstream of TLR is mediated by the MAP kinases ERK1 and ERK2. We found that EP strongly decreased the LPS-induced ERK 1 and ERK2 phosphorylation relative to the total level of the proteins, as early as 15 min and at 30 min post LPS-stimulation (Figure 5C). These results suggest that EP mediates at least some of its inhibitory effects by decreasing ERK phosphorylation.

### Ethyl Pyruvate Reverts the Metabolic Switch in LPS-Stimulated DCs at the Early and Late Activation Phases

**Figure 6:**
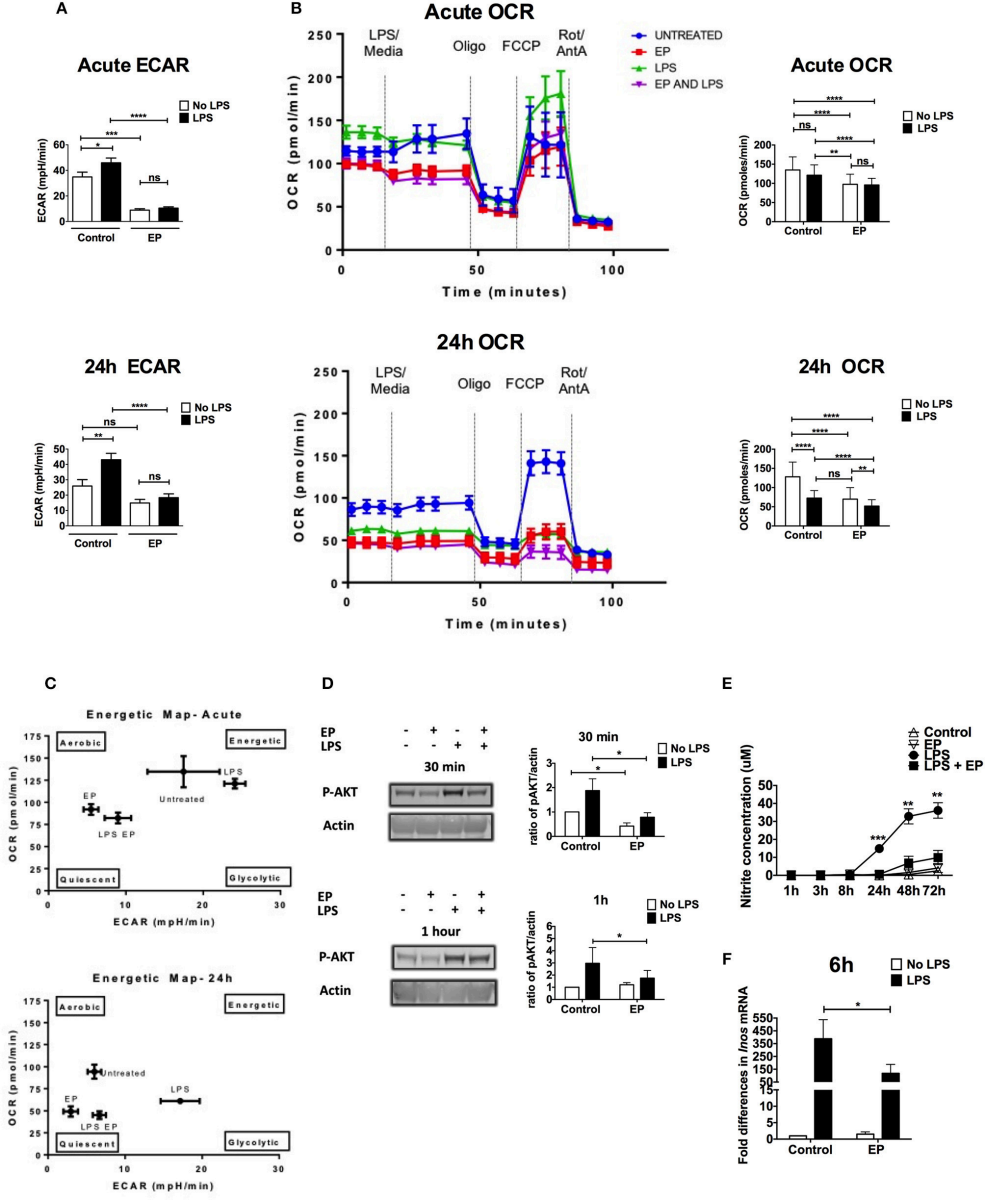
EP alters DC metabolism by targeting AKT and NO. We performed metabolic assays on DCs pre-treated with EP (10mM) 1 h before LPS (100 ng/ml) stimulation. **(A)** ECAR measurements at the acute time point (30min after LPS addition or at rate 7 of the assay, also equivalent to 30min after the beginning of the seahorse run; top) and 24 h post-stimulation (24 h post-LPS addition is equivalent to rate 7 of the assay or 30min after the beginning of the seahorse run; bottom), before the addition of mitochondrial inhibitors. Results are the average of 4 independent experiments (*n* = 4). **(B)** OCR measurements. DCs were stimulated with LPS for 30min (measurements taken at rate 7 of the assay, also equivalent to 30min after the beginning of the seahorse run; top) and 24 h (equivalent to rate 7 of the assay or 30min after the beginning of the seahorse run; bottom), and OCR was measured as a response to mitochondrial inhibitors: 1 μM oligomycin, 1.5 μM fluorocarbonyl cyanide phenylhydrazone (FCCP), and 100 nM rotenone plus 1 μM antimycin A. Representative OCR plots are shown on the left and the average of 4 independent experiments on the right (*n* = 4). **(C)** Energetic maps of control and treatments at the acute (30min; top) and the 24 h (bottom) time points. **(D)** Cells were harvested at 30min and 1 h after LPS stimulation and AKT phosphorylation measured by western blotting. Densitometry was analyzed by calculating the ratio of p-AKT to actin. Results are plotted as change in ratio from the untreated control and are shown as mean ± SEM of 3 independent experiments (*n* = 3). No change in trend when compared to total AKT. **(E)** Supernatants were analyzed for nitrite concentration as a proxy for nitric oxide levels using the Griess reagent kit. **(F)** Total RNA was extracted from DCs to determine *Inos* level of expression by qRT-PCR 6 h post-LPS using the Ct method. Values were normalized to cyclophillin and expressed as fold difference in mRNA from untreated cells. Results are shown as mean ± SEM and are from 6 independent experiments for nitrite levels (*n* = 6) and from 3 independent experiments for *Inos* level of expression (*n* = 3). Data in **(A,B,E)** was analyzed using the two-tailed unpaired Student's *t*-test and in **(B)** using the ratio paired two-tailed Student's *t*-test. *P < 0.05, **P < 0.01, ***P < 0.001, and ****P < 0.0001.

Inhibitors of immunometabolism have been shown to suppress DC activation (7, 8, 42). To investigate a possible effect of EP on the change in metabolism necessary to fuel DC activation, we performed metabolic assays on DCs pre-treated with EP then stimulated or not with LPS 1 h later using the XF-96 Extracellular Flux Analyzer (Seahorse) (6, 43). As previously published (7), LPS increased the extracellular acidification rate (ECAR), corroborating an increase in glycolysis, which was significant at both the acute (30 min) and 24 h time points (Figure 6A). EP significantly reduced the early LPS-induced increase in ECAR, which was maintained low at 24 h (Figure 6A). Furthermore, EP alone decreased the constitutive ECAR of resting DCs, suggesting a direct effect on the regulation of glycolysis (Figure 6A). When we assessed the oxygen consumption rate (OCR) of DCs as a measure of mitochondrial respiration at the acute time point (30 min post-LPS), we did not find any change in LPS-treated DCs as compared to untreated cells (Figure 6B top). However, LPS significantly reduced OCR when compared to control at 24 h (Figure 6B bottom), confirming the published observation that TLR agonists decrease mitochondrial oxygen consumption late after DC activation (6–8). Interestingly, EP did not rescue the LPS-induced OCR decrease but rather reduced it further (Figure 6B). EP alone also significantly decreased the constitutive OCR as compared to control at both time points (Figure 6B). This suggests that EP reduces mitochondrial respiration, either by inhibiting glycolysis or as an independent process. The energetic maps of control and treatments at 30 min and 24 h time points summarize our findings (Figure 6C), depicting how EP, alone and also despite LPS, shifts the cells to a quiescent state.

Further analysis of mitochondrial parameters showed that EP alone significantly decreased the constitutive ATP production and percent coupling efficiency at the acute time point (Supplemental Figures 2D,F), suggesting that EP can quickly reduce the energy levels and the mitochondrial Electron Transport Chain (ETC). During the late activation phase, EP reduced the constitutive basal and maximal respiration, proton leak, ATP production as well as non-mitochondrial respiration (Supplemental Figures 2A–D,G), suggesting that EP has a broad effect on the mitochondrial ability of DCs to aerobically consume oxygen. LPS on the other hand, increased proton leak and reduced % coupling efficiency at the acute phase (Supplemental Figures 2C,F) and suppressed all the mitochondrial parameters at 24 h as compared to control, confirming a strong suppression of mitochondrial respiration in the late DC activation phase (Supplemental Figures 2A–F). The lack of difference observed in percent spare capacity suggests that the differences in OCR may be due to differences in glycolysis (Supplemental Figure 2E). No change in non-mitochondrial respiration was detected with LPS at the acute time point, and the increase induced at 24 h was not statistically significant (Supplemental Figure 2G), possibly because of variations between experiments. EP significantly reduced the LPS-induced basal respiration and proton leak 30 min after stimulation (Supplemental Figures 2A,C), confirming a suppression of OXPHOS by EP, with and without LPS. EP also significantly decreased non-mitochondrial respiration in the presence of LPS (24 h time point), suggesting a suppression of the cytoplasmic oxidative processes stimulated by LPS, such as ROS production (Supplemental Figure 2G). These results confirm that LPS rapidly up-regulates glycolysis in DCs and shuts down mitochondrial respiration 24 h post-stimulation. EP attenuates glycolysis and OXPHOS, therefore blocking the metabolic switch required for DC activation.

### Ethyl Pyruvate Inhibits Glycolysis by Targeting AKT Phosphorylation During the Early DC Activation Phase

AKT phosphorylation is involved in the DC shift to glycolysis by directly inducing Hexokinase II phosphorylation, which is necessary for glycolysis up-regulation and DC activation (7). To determine whether EP affects this pivotal step in DC activation, we performed western blots on lysates from DCs pre-treated with EP/LPS and harvested at 30 min and 1 h after LPS stimulation. AKT phosphorylation was increased by LPS and significantly reduced with EP at the two tested time points (Figure 6D). The pattern did not change when compared to total AKT. These results suggest that EP attenuates glycolysis and hence early DC activation at least in part by decreasing TLR-induced AKT phosphorylation.

### Ethyl Pyruvate Attenuates the Metabolic Shift by Inhibiting Nitric Oxide Production During the Late DC Activation Phase

Nitric oxide (NO) is instrumental in suppressing mitochondrial respiration in the late DC activation phase and by extension in promoting glycolysis (11, 44). We measured nitrite concentration as a proxy to NO in culture supernatants of DCs pre-treated with EP followed by LPS 1 h later. We detected no nitrite in supernatants of LPS-stimulated DCs collected at 1, 3, and 8 h after activation. Nitrite levels were detectable starting 24 h and up to 72 h post-LPS and were strongly inhibited by EP at all time points (Figure 6E). Control supernatant or supernatant from cells treated with EP alone did not contain nitrite (Figure 6E). Concurrent with the nitrite data, EP significantly decreased the up-regulation of *Inos* transcripts 6 h after LPS stimulation (Figure 6F). Delayed EP treatment also suppressed nitrite levels induced by LPS, and decreased *Inos* transcript levels with a *p*-value approaching significance (Supplemental Figure 3). These results suggest that EP blocks the activation of signaling pathways downstream of TLR that lead to NO production, thereby attenuating the high glycolytic rate maintained by LPS in the late DC activation phase (6, 11).

### Ethyl Pyruvate Decreases the *in vivo* TLR-Induced DC Activation Without Affecting Their Viability

**Figure 7:**
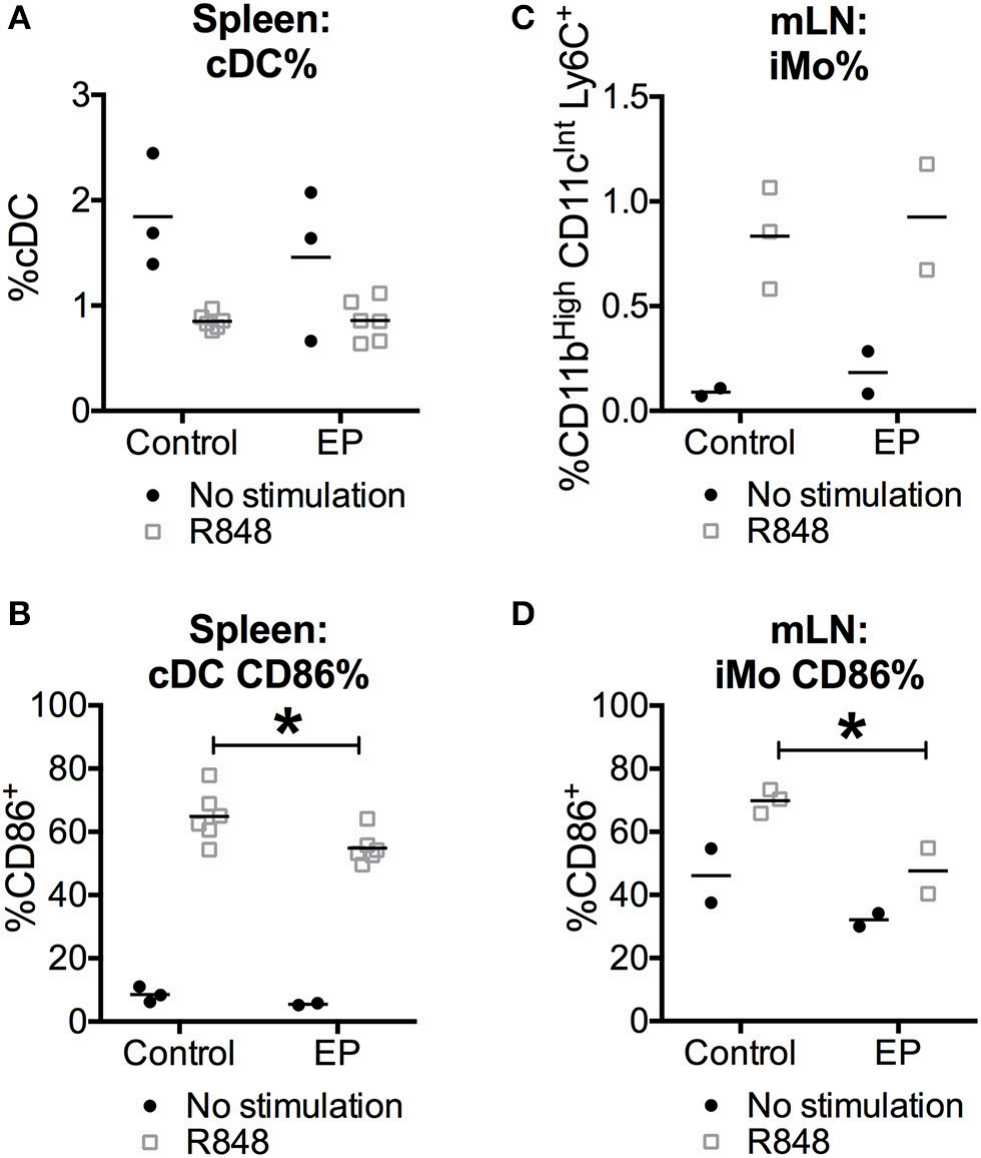
EP decreases the *in vivo* TLR-induced cDC activation without affecting their survival. C57BL/6 mice were injected i.p. with 80 mg/kg of EP in PBS (vehicle) 1 h before the injection of 30 μg/mouse of TLR7 ligand R848 in PBS (vehicle). EP was further administered again 4, 8, and 20 h after R848 stimulation. **(A,C)** Spleens and mesenteric lymph nodes (mLN) were stained for surface lineage markers to recognize cDCs (CD11c^Hi^, CD11b^Int^) and inflammatory monocytes (iMo, CD11c^Int^, CD11b^Hi^, and Ly6C^pos^) and **(B,D)** the costimulatory molecule CD86 24 h after the TLR stimulation. Results are expressed as mean and single results of 2 independent experiments (*n* = 3–6 for spleens and *n* = 2–3 for mLN). Data was analyzed using the two-way ANOVA and the Sidak correction for multiple comparisons test. *P*-values *P* < 0.05 were considered of statistical significance. *P < 0.05, **P < 0.01, ***P < 0.001, and ****P < 0.0001.

To test whether the suppressive effects of EP on DC activation *in vitro* were also observed *in vivo*, we harvested spleens and mesenteric lymph nodes (mLN), 24 h after C57BL/6 mice were injected with the TLR7 ligand R848, and received 80 mg/kg EP 1 h before as well as 4, 8, and 20 h after R848. We chose the TLR7 ligand R848 because it is a potent murine conventional DC (cDC) activator, which, unlike LPS (38), was not reported to induce DC death. We stained the cells *ex vivo* for surface lineage markers recognizing cDCs and inflammatory monocytes (iMo), two subsets of innate immune cells that are represented *in vitro* by the GM-CSF-DCs (1). We observed that the stimulation with R848 induced a decrease in percent splenic cDCs, which was unexpected because R848 was not previously associated with cell death, and an increase in iMo from mLNs. EP did not change the frequency of either innate subsets when comparing EP+R848 to R848-treated cells or EP-treated to unstimulated cells (Figures 7A,C). Moreover, we assessed cell activation status by analyzing the expression of surface CD86 and found that EP significantly decreased the percent of R848-induced CD86-positive cDCs (Figure 7B), reproducing the results observed with EP on the R848-mediated DC activation *in vitro* (Figure 4). In addition, EP was able to reduce R848-induced up-regulation of CD86 on iMo from mLNs (Figure 7D). These results were not due to cell death as no change in absolute cell numbers was caused by EP (data not shown), confirming once again that EP does not induce cell death. This data indicates that EP is able to directly affect cDCs *in vivo* by decreasing their activation without compromising cell viability, a promising finding for therapeutic purposes.

## Discussion

DC activation is vital to the initiation and maintenance of immune responses. It is however detrimental in conditions where inflammation and immunity are undesirable outcomes, such as in solid organ transplantation or in the autoimmune disease systemic lupus erythematosus. Targeting innate immunity, and in particular DCs, may promote less active or tolerogenic DCs. Herein we present the first report that EP suppresses DC activation and the metabolic reprogramming which follows it.

EP induced a potent shutdown of the response to LPS, with suppression of IL-12p70, TNF-α, and IL-6. Our findings are in agreement with reports showing that EP decreased pro-inflammatory cytokines in LPS-activated murine macrophages/RAW264.7 cells (24) and in rats with LPS-induced shock (27) and liver injury (19).

It is intriguing that EP significantly decreased the anti-inflammatory cytokine IL-10. We anticipated an increase in IL-10 to correlate with the decrease in IL-12p70 (45) and EP anti-inflammatory properties (21, 22, 24). The literature is controversial regarding the effect of EP on IL-10, which was increased by EP in rat serum upon LPS-induced shock (27), but was not altered in LPS-activated murine macrophages (21). We propose that IL-10 reduction in DCs is secondary to the EP-induced decrease in ERK phosphorylation, ERK being the master regulator of IL-10 secretion (46). Our results suggest that EP exerts a general level of suppression on DCs rather than a pro-inflammatory mediator-targeted suppression.

EP also significantly decreased the surface expression of the costimulatory molecules CD86, CD80, and CD40, thereby decreasing DC activation. In the absence of LPS, EP alone induced a small but significant increase in CD40 but not CD86 or CD80 surface expression. These cells however did not display characteristics of activation nor secreted any cytokine, confirming their resting status.

The suppressive effects of EP on DCs were not limited to LPS activation, because EP similarly decreased DC responses to TLR9 and TLR7 ligands, suggesting that EP can suppress TLR activation in general and possibly other stimulatory pathways.

DCs produce and respond to IFN-I when challenged with TLR ligands, and IFN-I play a role in DC activation and antigen presentation (40, 41, 47). The effect of EP on DCs was very rapid, targeting the transcription of the early gene *Ifnb* after 1 h and of the ISGs *Irf7, Mx1, Isg15* after 6 h of LPS stimulation. EP did not decrease *Cxcl10* RNA although it suppressed CXCL-10 protein expression. The discrepancy between mRNA and protein levels is not due to cell death as there were no significant differences in percentages of live cells nor in cell numbers among DCs activated in presence or absence of EP in the first 24 h. We propose that EP has a stronger effect on the protein rather than mRNA level for specific targets, as similarly shown with the glycolysis inhibitor 2-deoxyglucose (2-DG), possibly because energy demanding expansion of the ER and Golgi is required for protein translation (48). Other reports have also supported the idea that metabolic inhibition has greater effect at the post-transcriptional level, with the inhibition of protein translation more evident than the inhibition of RNA transcription (42). We have also previously published a similar discrepancy, as we found that estrogens, by enhancing the immunometabolism of DCs, increase CXCL10 protein but not RNA levels upon TLR stimulation (1). Likewise, in this paper, the effect of EP on the decrease in RNA level is significant but less striking than that on the proteins such as the IL-12p70 and IL-10 cytokines.

Our findings that EP partially inhibits signal 1 (MHC-II up-regulation), reduces signal 2 (costimulatory molecule up-regulation) and strongly suppresses signal 3 (cytokine production) and the IFN-I response in TLR-stimulated DCs, provide a biological explanation for the significantly decreased ability of DCs to stimulate an *in vitro* MLR, a strong T cell response, indicating that EP not only targets DCs phenotypically but also functionally. The significant decrease in DC function in the MLR demonstrates that EP is able to affect DC function in a setting that is representative of the polyclonal response occurring during physiological immune responses.

EP partially mediated its effects in LPS-stimulated DCs via an ERK 1/2-dependent, IKB-independent fashion. The lack of change in IKBa and IKBb degradation in EP-pre-treated DCs activated with LPS is in accordance with the report of Han et al. that EP does not target NF-kB translocation, but rather its binding to DNA by covalently modifying p65 Cys38 in RAW264.7 cells (30). We observed a reduction of ERK1/2 phosphorylation as early as 15 min post-LPS stimulation, in concordance with the report of Tsung et al. where EP reduced ERK phosphorylation among other MAPKs in a rat model of hepatic ischemia/reperfusion injury (19). We did not detect an effect of EP on p38 or JNK phosphorylation (data not shown), in agreement with studies where EP did not inhibit MAPKs in HEK293 cells (21), but in contrast to Ulloa et al. who reported an EP-mediated reduction in p38 phosphorylation in LPS-stimulated RAW264.7 cells (24). Therefore, EP affects MAPK phosphorylation differentially in distinct cell types. We propose that EP mediates its rapid effects on DCs at least in part by selectively targeting ERK phosphorylation.

Here we present the first report that EP alters DC metabolism. First, we confirm the results of Everts et al. (6, 42) that DCs rapidly up-regulate glycolysis, with a sharp rise in ECAR observed 30 min after LPS, which is maintained high after 24 h of stimulation. We also confirm the sustained OCR soon after LPS addition, showing that during the early phase of DC activation, mitochondrial respiration is still high, possibly to provide ATP and activate HK-II. The subsequent significant drop we found in OCR 24 h after LPS, is reminiscent of the mitochondrial shutdown due to NO production.

We found that EP suppressed early and late metabolic responses to activation. EP reduced the DC constitutive levels of glycolysis and abrogated its LPS-induced up-regulation, suggesting for the first time that EP is an inhibitor of glycolysis. EP could have been speculated to drive mitochondrial respiration by acting as the first substrate of the Krebs cycle, as it was suggested for the related compound methyl pyruvate (13, 29), although they did not directly measure ETC or OXPHOS. However, our findings show that EP alone significantly decreased OCR relative to control at early and late time points, and that in combination with LPS, further increased the shutdown occurring at 24 h, suggesting that EP is also an inhibitor of mitochondrial respiration. This inhibition may be due to an increased amount of pyruvate in the cell, resulting from the dissociation of EP into ethanol and pyruvate (32). Increased amounts of pyruvate may amplify the TCA cycle, and some TCA intermediates, such as citrate, might accumulate. If citrate is not fully used for fatty acid synthesis, the excess may induce a negative feedback inhibition by allosterically binding to the glycolytic enzyme phosphofructokinase (PFK), resulting in the blockade of glycolysis and by extension mitochondrial respiration (49). This could also partially explain the suppression of glycolysis induced by EP. Alternative explanations include a role for ethyl pyruvate as an inhibitory analog of pyruvate. It is not yet clear whether the effect of EP on the OCR in the cells is a direct metabolite effect or if it acts in a less direct manner through its effects on glycolysis or other mediators such as NO. The lack of difference observed in percent spare capacity would suggest that the differences in OCR may be due to differences in glycolysis.

In the early DC activation phase (up to 24 h post-activation), glycolysis up-regulation is mediated via the TBK1/IKKε/AKT axis, whereby AKT directly phosphorylates HK-II to activate it (42). HK-II is then able to translocate to the mitochondrial membrane where it has access to the Krebs cycle-generated ATP and promotes glycolysis (7, 12). The finding that EP decreased the LPS-induced phosphorylation of AKT provides a mechanism by which EP can target glycolysis, because a decrease in p-AKT levels, and by extension in HK-II activity, results in the drop in ECAR (7, 42, 50). Moreover, the decrease in p-AKT was observed as early as 30 min, endorsing the idea that the effect of EP is very fast and targets primarily the signaling pathways downstream of TLR, such as AKT and ERK. An interesting kinase that would be worth investigating in the future is mTOR. In fact, not only does mTOR promote glycolytic activation and anabolic pathway induction such as lipogenesis (12), it may also regulate dendritic cell long-term commitment to glycolysis via the induction of NO production by iNOS activation, to shut down mitochondrial respiration (6). Further investigation is warranted to address whether modulation of mTOR activation acts, together or not with TBK1, to affect AKT activation, the major mechanism that we have found for EP suppressive effects. The fact that delayed EP treatment also reduced DC activation after TLR stimulation, suggests that EP also affects DC metabolism downstream of the early signaling pathway, possibly directly at the glycolysis and respiration levels. The same conclusion is suggested by the fact that EP decreased ECAR, OCR and other measured mitochondrial parameters in absence of LPS stimulation, when AKT and MAPKs are not activated. The ability of EP to inhibit DC activation already in progress is an important requirement for a drug to be tested in chronic conditions, including autoinflammatory and autoimmune diseases.

Mitochondrial shutdown in the late DC activation phase (24 h post-TLR stimulation) has been proposed to be NO-mediated in iNOS-expressing DCs, leaving the cells with obligatory glycolysis up-regulation to survive (6). Concurrent with the literature, our LPS-stimulated DCs produced NO 24 h post-activation, the level of which increased until 72 h. EP drastically suppressed NO production at all time points, correlating with decreased *Inos* transcription 6 h after LPS stimulation. EP has been previously shown to suppress NO in various contexts (19, 21, 30). However, in our system, we correlate for the first time the EP-induced inhibition of NO with the effect on DC metabolism. NO suppression by EP can explain the persistent absence of glycolysis up-regulation upon LPS stimulation and suggests that the 24 h decrease in OXPHOS is a direct effect of EP rather than LPS. Furthermore, the effect of EP on iNOS expression and nitrite levels might hint to the extent of cells dependence on NO attenuation for survival and maturation at the 24 h time point. However, we exclude that DCs depend on NO attenuation for the EP suppression of their maturation because all fatty acid and protein synthesis would have already occurred during the early activation stage prior to NO production (7, 8). As for their survival, Everts et al. have already reported experiments with iNOS inhibitor in LPS-activated DCs and analyzed for cell activation markers, cytokine production as well as viability 24 h post-stimulation. Dendritic cells with iNOS inhibitor were able to be normally activated, secrete cytokines and did not depend on NO for their survival in a milieu containing glucose (6).

It was recently shown that DCs can use intracellular storages of glycogen to sustain cellular metabolism in presence of low extracellular glucose concentrations (5 mM) (51). We performed all our experiments in IMDM medium containing 4.5 g/L of glucose (25 mM) that is more than twice the amount (10 mM) present in RPMI medium used in other reports (52, 53). Therefore, EP inhibits DC immunometabolism in presence of sufficient glucose amounts to support DC activation, suggesting that the immunometabolism blockade is independent of the extracellular glucose and intracellular glycogen.

EP decreased ECAR and OCR to levels that do not allow activation but do not induce cell death. Moreover, EP rescued DCs from the LPS-induced cell death 72 h post-stimulation. Pearce et al. have previously reported that with time, LPS-activated DCs die in culture due to glucose consumption and that increased death is observed with cells under restricted glucose conditions, while adequately supplementing DCs with glucose, or inhibiting mTOR, sustains viability (8, 11). Our finding that EP maintained survival of DCs after 72 h of stimulation is suggestive of glucose consumption reduction due to a blockade in the signaling pathways downstream of TLR. It is also reminiscent of the anti-cell death properties of EP such as the inhibition of apoptosis, necrosis and autophagy in animal models of tissue damage (19, 54). In fact, when performing our cell death and metabolism experiments, we observed no significant differences in percentages of live cells and absolute cell numbers between DCs activated in presence or absence of EP, except with 20 mM of EP. The cell death induced by 20 mM of EP could be due to an excessive suppression of DC immunometabolism. Alternatively, an increased electrophile reactivity overriding EP ROS-scavenging properties, may lead to a highly-oxidized milieu and thus toxicity (13).

The *in vivo* injection of EP in mice significantly decreased the activation of cDCs upon TLR stimulation. We used the TLR7 ligand R848 based on its potency as a cDC activator and the assumption that it does not compromise cell viability. Our finding that R848 decreased the percentage of cDCs may suggest otherwise, or it may be due to a shift in the expression of the differentiation markers used to gate the cDCs. Future experiments will better characterize the *in vivo* effects of R848 on innate subsets and differences with LPS or other TLR ligands. Important for our initial hypothesis, is the result that EP decreased the activation of cDCs upon TLR stimulation, which confirms *in vivo* our main *in vitro* results. Moreover, the inhibitory effects of EP were also significant in the inflammatory monocytes, another innate immune subset involved in pro-inflammatory responses, further supporting a suppressive role for EP on the activation of innate immune cells.

In conclusion, our results support our discovery that EP inhibits DC activation by directly affecting their energy metabolism. This is the first report showing that EP inhibits most of the DC biological responses to TLR stimulation in an ERK-, AKT-, and NO-dependent manner, altering the metabolic reprogramming necessary for DC activation through the inhibition of glycolysis and mitochondrial respiration. Furthermore, the direct effect of EP on cDCs *in vivo* and the decrease in their activation without compromising cell viability, represents a promising finding for putative therapeutic purposes.

## Author Contributions

MC and SG: conceptualization and methodology; MC, PS, CQ, and ML: formal analysis; MC, RWC, PS, JM, ML, and CQ: investigation; MC: writing-original draft; MC, RWC, PS, CQ, ML, JM, TE, WK, RC, and SG: writing-review and editing; SG, TE, and RC: funding acquisition; SG, TE, WK, and RC: resources; SG: supervision.

### Conflict of Interest Statement

The authors declare that the research was conducted in the absence of any commercial or financial relationships that could be construed as a potential conflict of interest.
