# Supplementary material for: Ethyl Pyruvate Modulates Murine Dendritic Cell Activation and Survival Through Their Immunometabolism
Source: Front Immunol. 2019 Jan 28;10:30. doi: 10.3389/fimmu.2019.00030 (PMC6362406; doi:10.3389/fimmu.2019.00030)
Supplement: Supplementary file 1 [file Data_Sheet_1.pdf]

# Supplemental Information

# Ethyl pyruvate modulates murine dendritic cell activation and survival through their immunometabolism


**Marita Chakhtoura[1,7], Robert W. Chain[1], Priscila Y. Sato[3,4,8], Connie C. Qiu[1], Michael H. Lee[1], Joseph J. Meissler[2,5], Toby K. Eisenstein[2,5], Walter J. Koch[3,4], Roberto Caricchio[6], and Stefania Gallucci[1,9,*]**

[1]Laboratory of Dendritic Cell Biology, Department of Microbiology-Immunology, Lewis Katz School of Medicine, Temple University, Philadelphia, PA 19140, USA.

[2]Department of Microbiology-Immunology, Lewis Katz School of Medicine, Temple University, Philadelphia, PA 19140, USA.

[3]Center for Translational Medicine, Lewis Katz School of Medicine, Temple University, Philadelphia, PA 19140, USA.

[4]Department of Pharmacology, Lewis Katz School of Medicine, Temple University, Philadelphia, PA 19140, USA.

[5]Center for Substance Abuse Research, Lewis Katz School of Medicine, Temple University, Philadelphia, PA 19140, USA.

[6]Division of Rheumatology, Department of Medicine, Lewis Katz School of Medicine, Temple University, Philadelphia, PA 19140, USA.

[7]Present address Department of Medicine, Division of Infectious Diseases and HIV Medicine, Drexel College of Medicine, Philadelphia, PA 19102

[8] Present address Department of Pharmacology and Physiology, Drexel University College of Medicine, Philadelphia, PA 19102, present.

[9]Lead Contact

**Supplemental Figure 1.**

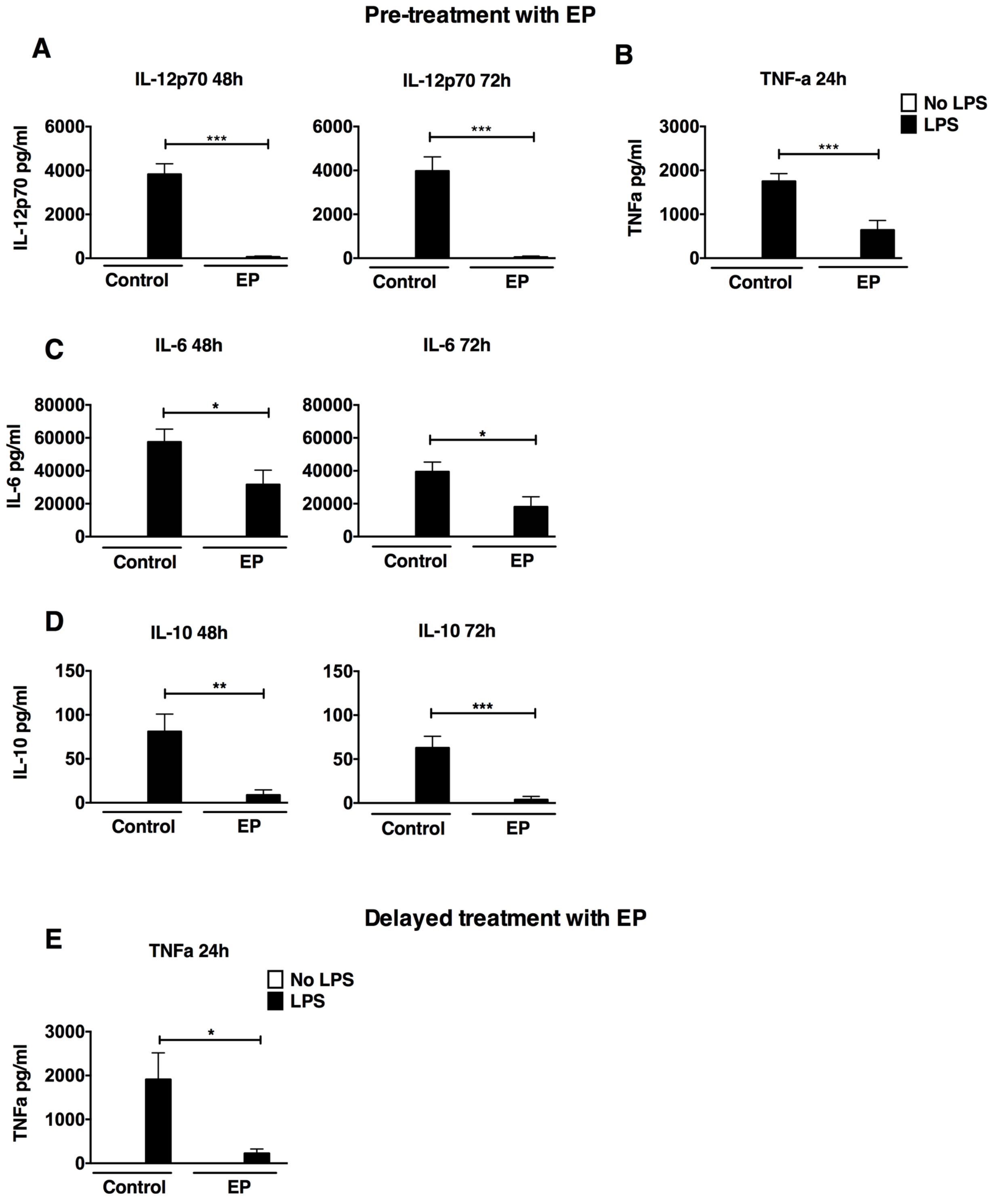

**Supplemental Figure 1. Ethyl pyruvate exerts a general level of cytokine production suppression in LPS-stimulated DCs.** We cultured DCs in presence of GMCSF for 6-7 days then stimulated them with 10mM EP in presence or absence of 100ng/ml LPS. EP treatments were administered 1h prior **(A-D)** or after **(E)** TLR stimulation. Supernatants were collected at 24h, 48h and 72h post-LPS (**A-D**) and 24h post-EP (**E**) and analyzed for the levels of the indicated cytokines by ELISA. Results are from at least 3 independent experiments (n=3) and are expressed as mean ± SEM. Data was analyzed using unpaired two-tailed Student's t-test with P-values $P<0.05$ considered of statistical significance. * $P<0.05$, ** $P<0.01$, *** $P<0.001$ and **** $P<0.0001$.

**Supplemental Figure 2.**

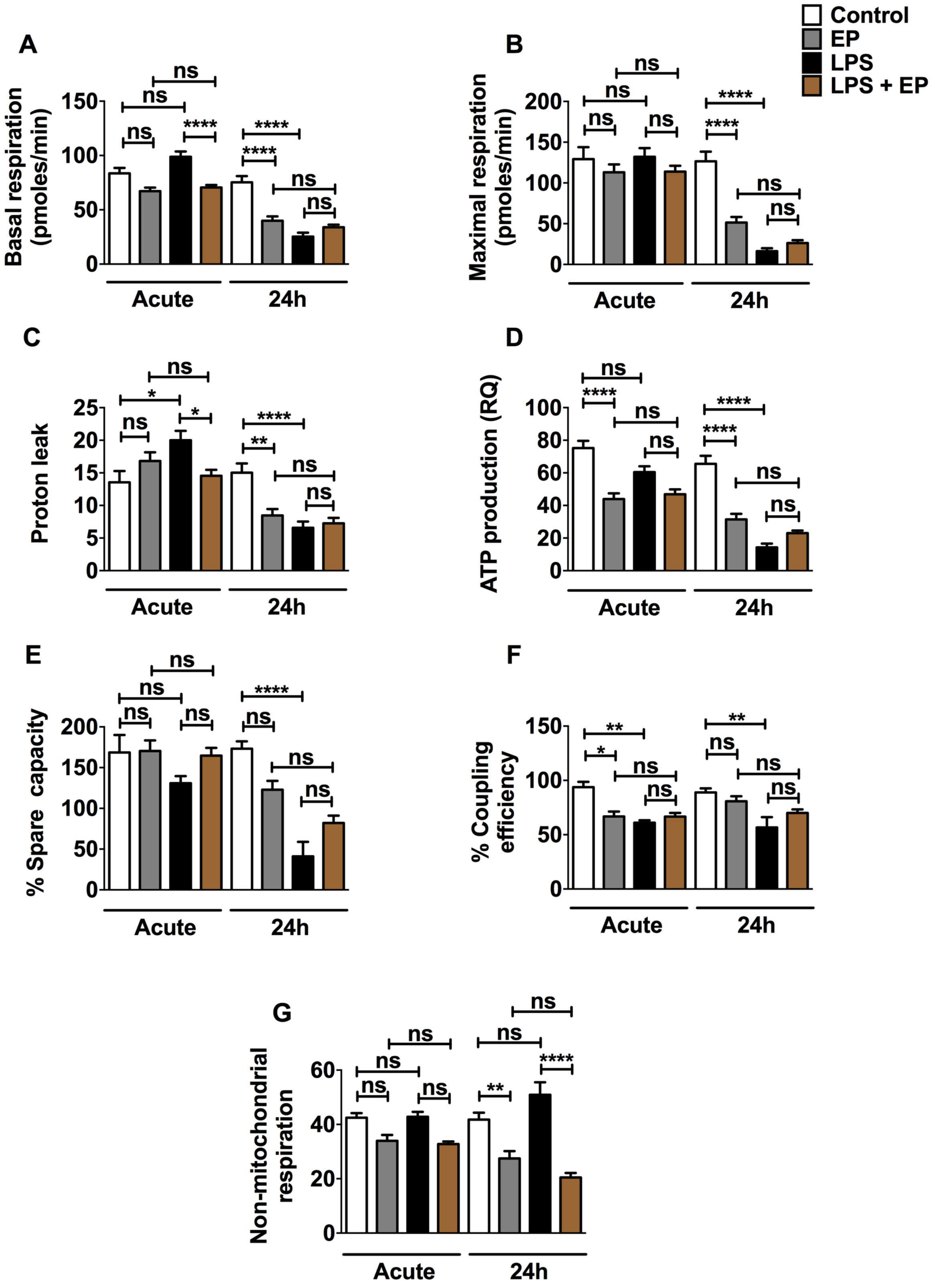

**Supplemental Figure 2. Ethyl pyruvate alters the LPS-induced shift in metabolism.** We performed metabolic assays on DCs pre-treated with EP (10mM) 1 hour before LPS (100ng/ml) stimulation and measured their response to mitochondrial inhibitors: 1μM oligomycin, 1.5μM fluorocarbonyl cyanide phenylhydrazone (FCCP) and 100 nM rotenone plus 1μM antimycin A. Illustrated parameters are **(A)** Basal respiration **(B)** maximal respiration **(C)** proton leak **(D)** ATP production **(E)** percent spare capacity **(F)** percent coupling efficiency and **(G)** non-mitochondrial respiration**.** Results are the average of 4 independent experiments (n=4) and are shown as mean ± SEM. Data was analyzed using One-way ANOVA followed by the Bonferroni multiple comparisons test. P-values P<0.05 were considered of statistical significance. * P<0.05, ** P<0.01, *** P<0.001 and **** P<0.0001.

**Supplemental Figure 3.**

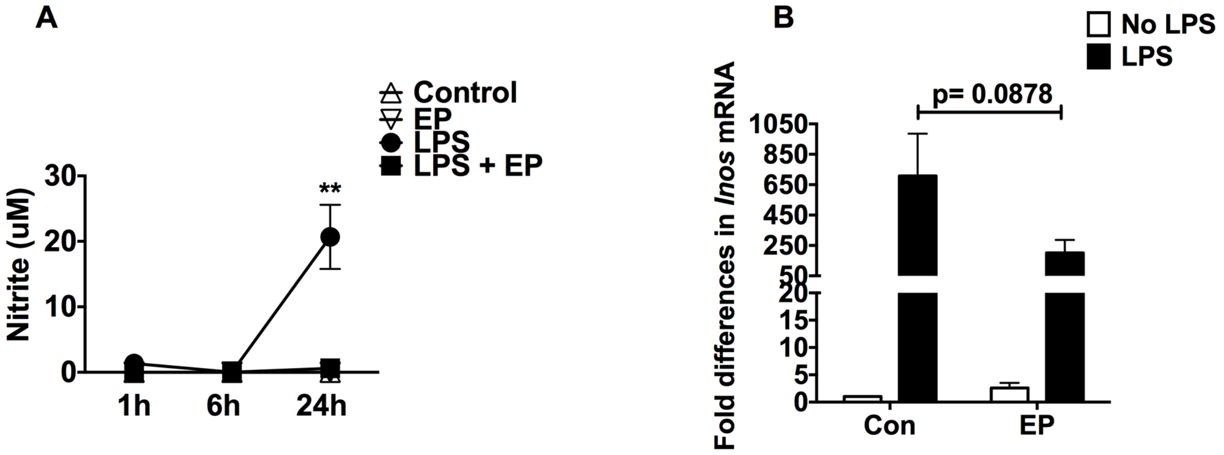


**Supplemental Figure 3. Delayed ethyl pyruvate treatment inhibits NO production at the later phase of DC activation, thereby attenuating glycolysis. (A)** We treated DCs in culture with 100ng/ml LPS followed by 10mM EP an hour later. Supernatants were collected at 1, 6 and 24h post-EP and analyzed for nitrite concentration as a proxy for nitric oxide levels using the Griess reagent assay. (B) Total RNA was extracted from cells to determine *Inos* level of expression by qRT-PCR 6h post-EP using the Ct method. Values were normalized to cyclophillin and expressed as fold difference in mRNA from untreated cells. Results are shown as mean ± SEM and are from 4 independent experiments (n=4). Data was analyzed using unpaired two-tailed Student's t-test with P-values P<0.05 considered of statistical significance. * P<0.05, ** P<0.01, *** P<0.001 and **** P<0.0001.

**STAR Methods**

## EXPERIMENTAL MODEL AND ANIMAL DETAILS

*Mice*

Female C57BL/6 (B6) (Cat no. 000664; RRID:IMSR_JAX000664) and C3HeB/FeJ (Cat no. 000658; RRID:IMSR_JAX000658) mice were purchased from the Jackson Laboratory (Bar Harbor, ME, USA) and maintained in our colony in accordance with the guidelines of the Institutional Animal Care and Use Committees of Lewis Katz School of Medicine at Temple University, a member of the American Association for the Accreditation of Laboratory Animal Care-accredited facilities. All mice were housed in autoclaved microisolator cages on ventilated racks and fed sterile rodent chow and water. No more than 5 mice were in the same cage. The facility is a pathogen-free environment with stable temperature, humidity and a 12-hour light and 12-hour dark cycle. Full personal protective equipment was used when handling the animals. Mice were used between 6 and 12 weeks of age. C56BL/6 mice were used to generate DCs from their bone marrow cells. Spleens from C3HeB/FeJ female mice were used as the source of responders in the mixed lymphocyte reaction (MLR) assays.

## METHOD DETAILS

*In vitro bone marrow-derived DC cultures*

Bone marrow precursor cells were flushed from femurs and tibias of B6 mice and differentiated into DCs in presence of granulocyte macrophage-colony stimulating factor (GM-CSF) as previously described (Sriram et al., 2012; Sriram et al., 2014). The cells were seeded as one million per milliliter of complete Iscove's modified Dulbecco's medium (IMDM) (with 10% FBS,

100U/ml penicillin/streptomycin, 200mM L-glutamine and gentamicin) enriched with 1% GM-CSF conditioned medium from a GM-CSF-producing cell line in 24 well plates (GM-CSF collected from the supernatant of a B7H1-GM cell line for GM-CSF conditioned medium). On day 2 of the culture, 1ml of fresh medium was added to each well. Starting day 5 and until the day the culture was used, 1ml per well was replaced with fresh medium every day. The differentiated DCs were stimulated on day 6 or 7 with ethyl pyruvate (EP) 98% (Sigma-Aldrich; Cat no. E47808) diluted in IMDM, and/or 100ng/ml LPS from *Escherichia coli* (Sigma-Aldrich; Cat no. L3755). EP was administered to the cells one hour prior to LPS. In EP dose titration experiments, EP was added to the cells 1h before LPS at the final concentrations of 1mM, 3.4mM, 5mM, 10mM or 20mM. Where indicated, DCs were stimulated with R848 (1ug/ml) (Invivogen; Cat no. tlrl-r848-5) or CpG B (10ug/ml) (Invivogen; Cat no. tlrl-1826) as TLR7 and 9 ligands respectively. In select experiments, EP treatment was delayed and followed LPS stimulation by 1 hour.

*Assessment of cell viability by flow cytometry*

The generated DCs were harvested at 8, 24, 48 and 72h post-LPS stimulation and stained with Annexin V (BD Bioscience; Cat. no. 556419; RRID:AB_2665412) and 7-AAD (BD Bioscience; Cat no. 559925) to determine cell viability. Briefly, harvested cells were stained with Annexin V in Annexin V-binding buffer for 15 minutes before the addition of 7-AAD. Cells were analyzed on a FACSCanto flow cytometer (BD Bioscience) and FlowJo software was utilized for data analysis (Tree Star, Ashland, OR, USA).

*Assessment of dendritic cell activation by flow cytometry*

DCs were harvested at 24 and 48h following LPS stimulation and analyzed by flow cytometry for the expression of surface costimulatory markers as well as MHC molecules. Briefly, cells were

incubated with FcγR blocker (purified anti-mouse CD16/CD32, clone 93; Biolegend; Cat no. 101310; RRID:AB_2103871) in PBS with 10% FBS for 15 minutes. They were then stained with fluorochrome-conjugated antibodies against DC surface markers for 30 minutes on ice. The antibodies used were directed against mouse CD11c (N418) (Cat no. 17-0114-82; RRID:AB_469346), CD86 (GL-1) (Cat no. MA1-10300; RRID:AB_11153707) and MHC-II (M5/114.15.2) (Cat no.13-5321-82; RRID:AB_466662) and were all purchased from eBioscience (San Diego, CA). Anti-MHC-II was a biotinylated antibody, hence streptavidin-PerCPCy5.5 (eBioscience, Cat no. 45-4317-82; RRID:AB_10311495) was added to the cells in a secondary step to stain positive DCs. Anti-CD11b (M1/70) (Cat no. 561098; RRID:AB_2033994), anti-CD40 (HM40-3) (Cat no. 553723; RRID:AB_395008) and anti-CD80 (16-10A1) (Cat no. 561955; RRID:AB_10892805) were all from BD Biosciences (San Jose, CA), while anti-MHC-I (H2kb) (28-8-6) (Cat no. 114608; RRID:AB_313599) was from Biolegend (San Diego, CA). Cells were fixed in 2% paraformaldehyde in PBS and analyzed on a FACSCanto flow cytometer (BD Bioscience) with FlowJo software (Tree Star, Ashland, OR, USA). In experiments where EP was added after LPS, flow cytometry was performed 24h after EP treatment.

*Measurement of cytokine levels by ELISA*

Supernatants were collected from DC cultures at 8, 24, 48 and 72h post-LPS stimulation for the measurement of IL-12p70, TNF-α, IL-6 and IL-10 levels using ELISA kits (BD Pharmingen) according to the manufacturer's protocol. ELISA plates were coated overnight with capture antibody. Plates were blocked with 10% FBS in PBS before cytokine standards and sample supernatants were added in duplicates. Optical densities were measured at 450nm and results analyzed with SoftMax Pro software (Molecular Devices Corporation, Sunnyvale, CA). For

CXCL-10 levels, the R&D ELISA kit was used according to the manufacturer's protocol. For experiments with CpG, R848 and cultures where EP was used post-TLR stimulation, 6h and 24h supernatants post-treatment were collected for ELISA.

*Gene expression quantification by q RT- PCR*

Gene expression of *in vitro* DCs was analyzed by quantitative reverse transcription PCR (qRT-PCR) using Taqman probes. Total RNA was extracted from cells harvested 1h and 6h after LPS stimulation using the Quick-RNA MiniPrep kit (Zymo Research; Cat no. R1055) according to the manufacturer's protocol. The extracted RNA was reverse transcribed using the High Capacity cDNA RT kit (Applied Biosystems; Cat no. 4368814). Pre-made Taqman primers and probes purchased from Applied Biosystems were used to assess the level of expression of *Ifnb*, *Mx1*, *Isg15*, *Irf7*, *Cxcl10* and *Inos*. Cyclophillin (*Cyc*) was used as the housekeeping gene for normalization. The Ct method of relative quantification of gene expression was used for analysis and the normalized Ct values (against cyclophillin) were calibrated against the control sample (untreated DCs) in each experiment. In experiments where EP was used post-LPS stimulation, qRT-PCR analysis was performed 1 and 6h after EP treatment.

*Western blot analysis*

Thirty micrograms of denatured total lysate were used in sodium dodecyl sulfate-polyacrylamide gel electrophoresis (SDS-PAGE) for Western Blot. Proteins were transferred onto polyvinylidene fluoride (PVDF) membranes and blocked with 5% milk or Bovine serum albumin (BSA) in Tris Buffered Saline +0.1% Tween-20 (TBST). Membranes were incubated overnight at 4°C with primary antibodies against phosphorylated and total ERK 1/2 (p-ERK: Cat no. 4370;

RRID:AB_2315112; Total ERK: Cat no. 9107; RRID:AB_10695739), p38 (p-p38: Cat no. 9211; RRID:AB_331641; Total p38: Cat no. 9212; RRID:AB_330713), JNK 1/2 (p-JNK: Cat no. 9251; RRID:AB_331659; Total JNK: Cat no. 9252; RRID:AB_2250373), AKT (p-AKT: Cat no. 9271; RRID:AB_329825; Total AKT: Cat no. 9272; RRID:AB_329827) as well as total IKBa (Cat no. 4814; RRID:AB_390781) (Cell Signaling) (diluted in 5% BSA in TBST) and primary antibody against total IKBb (Santa Cruz Biotechnology; Cat no. sc-945; RRID:AB_631696) (diluted in 5% milk in TBST). Rabbit anti-mouse (Abcam; Cat no. ab5694; RRID:AB_2223021) and mouse anti-mouse (Santa Cruz Biotechnology; Cat no. sc-8432; RRID:AB_626630) actin were used as loading controls where appropriate. Mouse anti-mouse GAPDH (EMD Millipore; Cat no. MAB374; RRID:AB_2107445) was also used as an extra loading control when probing for ERK proteins. Membranes were then incubated with IR Dye 800 goat anti-rabbit (Cat no. ABIN2169623) and IR Dye 680 donkey anti-mouse (Cat no. ABIN2169633) (LI-COR Biosciences) diluted in blocking buffer plus 0.1% Tween-20. Proteins were visualized by scanning the membranes on an Odyssey Infrared Imaging System (LI-COR Biosciences) in both 700nm and 800nm channels.

*Mixed lymphocyte reaction assay*

Single cell suspensions were prepared from spleens of C3HeB/FeJ female mice and plated on large petri dishes to allow the Antigen Presenting Cells (APCs) to adhere to the plastic. After 90 minutes of incubation, non-adherent cells were harvested and counted as APC-depleted responders. DCs from B6 female mice were generated in culture as described above and treated on day 6 with or without 10mM EP in the presence or absence of 100ng/ml LPS 1h later. After 24h of stimulation, DCs were washed and added to allogeneic APC-depleted C3HeB/FeJ female splenocytes (800,000 cells per well) in a DC:splenocyte ratio of 1:30. Cells were plated in RPMI-

1640 + 10% FBS+ 0.05mM 2-Mercaptoethanol and 50ug/ml gentamycin. Each condition of DC:splenocyte was run in quadruplicate. B6 DCs alone from each condition and C3HeB/FeJ splenocytes alone served as controls. Plates were incubated at 37°C for 48h. Then cells were pulsed with 1uCi of $^3$H thymidine for 18 h, harvested and counted with a Tri Carb liquid scintillation counter (PerkinElmer, Waltham, MA) for radioactivity in counts per minute (cpm). Incorporation of $^3$H thymidine is a measure of splenocyte proliferation post-interaction with the non-syngeneic DCs. Cpm recorded for every condition was subtracted from that of the appropriate control DCs. Stimulation Index was calculated using the formula cpm of a sample / cpm of splenocytes incubated with DCs left untreated in the absence of EP and LPS.

*Metabolism assays*

Real time analysis of oxygen consumption rate (OCR) and extracellular acidification rate (ECAR) was performed using the Seahorse X-96 metabolic extracellular flux analyzer (Seahorse Bioscience). Metabolism assays were run and ECAR and OCR measurements were recorded at an acute (30 min) and 24h time points. For the acute time point, plots were generated based on the values recorded at rate 7 of the assay, which is equivalent to 30 min after addition of LPS or medium for the cells that did not receive LPS. This also coincides with 30 min after the beginning of the Seahorse run. As for the 24h time point, plots were generated based on the values recorded 24h following the addition of LPS or medium for the cells that did not receive LPS. This time point is equivalent to the rate 7 of the assay, coinciding with 30 min after the beginning of the run. For the 24h time point, DCs were generated in culture as previously described and either left untreated, treated with EP (10mM) and/or LPS (100ng/ml) for 24h. The cells were then harvested and plated at a density of 70,000 cells per 70ul per well of XF-96 culture plates previously coated

with poly-d-lysine for adherence. Cells were then washed in XF assay medium (unbuffered RPMI, 10mM glucose, 10% FCS, 100 U/mL penicillin/streptomycin, 2mM L-glutamine, 1% GM-CSF supernatant) and analyzed for OCR and ECAR in response to 1μM oligomycin, 1.5μM fluoro-carbonyl cyanide phenylhydrazone (FCCP) and 100 nM rotenone plus 1μM antimycin A as part of the Seahorse XF Cell Mito Stress Test kit (Agilent, Santa Clara, CA). The use of the Mito Stress Test Kit is an acceptable method for ECAR measurement (Nicholas et al., 2017; Tan et al., 2015; Wang et al., 2013), since it is able to measure the acidification caused by the $H^+$ ions in the milieu. For the acute time point, DCs were harvested and treated directly in the XF-96 culture plates.

*Nitric oxide quantification*

Nitrite concentration was measured as a proxy for nitric oxide (NO) in DC culture supernatants collected at 1, 3, 8, 24, 48 and 72h post-LPS stimulation. For experiments with delayed EP treatment, supernatants were analyzed at 1, 6 and 24h after EP treatment. A colorimetric assay using the Griess reagent (Acros Organics; Cat no. AC328670500) was utilized to measure nitrite levels in uM according to the manufacturer's protocol. Absorbance was measured at 550nm and a standard curve was generated.

*In vivo EP injection and spleen and lymph node cell staining*

C57BL/6 mice were injected i.p. with 80mg/kg of EP in 200ul PBS or PBS vehicle alone, one hour before the injection of 30 ug/mouse of TLR7 ligand R848 in 200ul PBS or PBS vehicle alone. EP was further administered again 4, 8 and 20h after R848 stimulation. Spleens and mesenteric lymph nodes (mLN) were harvested 24h post-R848 stimulation and physically disrupted with a 70um cell strainer to prepare single cell suspensions. Spleens were further digested with DNase I from bovine pancreas (Sigma-Aldrich; Cat no. D4527) and Collagenase Type IV (Worthington

Biochemical Corporation; Cat no. LS004186) treatment followed by Ammonium-Chloride-Potassium (ACK) red blood cell lysis buffer (VWR; Cat no. 118-156-101). Viability of splenic and mLN cells was assessed by either staining with fixable viability dye eFluor780 (eBioscience; Cat no. 65-0865-14) prior to the addition of primary antibody panels or with 7-AAD. Cells were incubated with purified rat anti-mouse CD16/CD32 monoclonal antibody (clone 2.4G2; BD Biosciences; Cat no. 553142; RRID:AB_394657) prior to the addition of fluorochrome-conjugated antibodies against conventional DC/inflammatory monocyte surface markers. Cells were stained with eBioscience rat anti-mouse CD11c (N418), CD86 (GL-1) and Ly-6C (HK1.4) (Cat no. 25-5932-82; RRID:AB_2573503) as well as BD Biosciences rat anti-mouse CD11b (M1/70). Cells not stained with 7-AAD were fixed with 1% paraformaldehyde. Samples were acquired on a FACSCanto Cytometer then analyzed with FlowJo.

## QUANTIFICATION AND STATISTICAL ANALYSIS

Prism 6 (GraphPad software, San Diego, CA, USA) was used for data analysis. Means and Standard Errors (Mean ± SEM) were calculated by averaging results from independent experiments. Throughout the manuscript, "n" refers to independent cell cultures from individual mice, when each culture may have two-three biological replicate. Statistical significance was determined using unpaired two-tailed Student's t-test or ratio paired two-tailed Student's t-test for comparison between two groups. One-way ANOVA was used as appropriate for multiple comparisons in one group followed by the Bonferroni multiple comparisons post-hoc correction test. Two-way ANOVA was used as appropriate for multiple comparisons between two groups followed by the Sidak multiple comparisons post-hoc correction test. P-values of $p < 0.05$ (marked

in the figures as * P<0.05, ** P<0.01, *** P<0.001 and **** P<0.0001) were considered significant.

**STAR methods:**

**Key Resource Table**

| REAGENT or RESOURCE | SOURCE | IDENTIFIER |
|---|---|---|
| **Antibodies** | | |
| LEAF purified rat monoclonal anti-mouse CD16/CD32 (clone 93) | Biolegend | Cat # 101310; RRID:AB_2103871 |
| APC Armenian hamster monoclonal anti-mouse CD11c (clone N418) | eBioscience | Cat # 17-0114-82; RRID:AB_469346 |
| FITC rat monoclonal anti-mouse CD86 (clone GL-1) | eBioscience | Cat # MA1-10300; RRID:AB_11153707 |
| Biotinylated rat monoclonal anti-mouse MHC-II (I-A/I-E) (clone M5/114.15.2) | eBioscience | **Cat #** 13-5321-82; RRID:AB_466662 |
| Streptavidin-PerCPCy5.5 | eBioscience | Cat # 45-4317-82; RRID:AB_10311495 |
| PeCy7 rat monoclonal anti-mouse CD11b (clone M1/70) | BD Biosciences | Cat # 561098; RRID:AB_2033994 |
| FITC Armenian hamster monoclonal anti-mouse CD40 (clone HM40-3) | BD Biosciences | Cat # 553723; RRID:AB_395008 |
| PE Armenian hamster monoclonal anti-mouse CD80 (clone 16-10A1) | BD Biosciences | Cat # 561955; RRID:AB_10892805 |
| PE mouse monoclonal anti-mouse MHC-I (H-2K$^b$/H-2D$^b$) (clone 28-8-6) | Biolegend | Cat # 114608; RRID:AB_313599 |
| Purified rat monoclonal anti-mouse CD16/CD32 (clone 2.4G2) | BD Biosciences | Cat # 553142; RRID:AB_394657 |
| PeCy7 Ly-6C rat monoclonal anti-mouse antibody (clone HK1.4) | eBioscience | Cat # 25-5932-82; RRID:AB_2573503 |
| Phospho-p44/42 MAPK (Erk1/2) (Thr202/Tyr204) rabbit anti-mouse monoclonal antibody (clone D13.14.4E) XP® | Cell Signaling | Cat # 4370; RRID:AB_2315112 |

| | | |
|---|---|---|
| Total p44/42 MAPK (ERK1/2) mouse anti-mouse monoclonal antibody (clone 3A7) | Cell Signaling | Cat # 9107; RRID:AB_10695739 |
| Phospho-SAPK/JNK (Thr183/Tyr185) rabbit anti-mouse polyclonal antibody | Cell Signaling | Cat # 9251; RRID:AB_331659 |
| Total SAPK/JNK rabbit anti-mouse polyclonal antibody | Cell Signaling | Cat # 9252; RRID:AB_2250373 |
| Phospho-p38 MAPK (Thr180/Tyr182) rabbit anti-mosue polyclonal antibody | Cell Signaling | Cat # 9211; RRID:AB_331641 |
| Total p38 MAPK rabbit anti-mouse polyclonal antibody | Cell Signaling | Cat # 9212; RRID:AB_330713 |
| Total IkBa (Amino-terminal antigen) mouse anti-mouse monoclonal antibody (clone L35A5) | Cell Signaling | Cat # 4814; RRID:AB_390781 |
| Total IkBb (C-20) rabbit anti-mouse polyclonal antibody | Santa Cruz Biotechnology | Cat # sc-945; RRID:AB_631696 |
| Phospho-AKT (Ser473) rabbit anti-mouse polyclonal antibody | Cell Signaling | Cat # 9271; RRID:AB_329825 |
| Total AKT rabbit anti-mouse polyclonal antibody | Cell Signaling | Cat # 9272; RRID:AB_329827 |
| Rabbit anti-mouse polyclonal anti-alpha smooth muscle Actin | Abcam | Cat # ab5694; RRID:AB_2223021 |
| Mouse anti-mouse monoclonal Actin (C-2) antibody | Santa Cruz Biotechnology | Cat # sc-8432; RRID:AB_626630 |
| Mouse anti-mouse monoclonal GAPDH antibody (clone 6C5) | EMD Millipore | Cat # MAB374; RRID:AB_2107445 |
| Goat anti-rabbit IgG (heavy and light chain) polyclonal antibody (IRDye800CW) | LI-COR Biosciences | Cat # ABIN2169623 |
| Donkey anti-mouse IgG (heavy and light chain) polyclonal antibody (IRDye680RD) | LI-COR Biosciences | Cat # ABIN2169633 |
| **Chemicals, Peptides, and Recombinant Proteins** | | |
| FITC Annexin V | BD Biosciences | Cat # 556419; RRID:AB_2665412 |
| Annexin V binding buffer | BD Biosciences | Cat # 556454 |

| | | |
|---|---|---|
| 7-AAD | BD Biosciences | Cat # 559925 |
| Corning™ Cellgro™ Iscove's modification of DMEM medium | Fisher Scientific | Cat # MT10016CV |
| Ethyl pyruvate 98% | Sigma-Aldrich | Cat # E47808 |
| LPS from (*Escherichia coli* Serotype O26:B6) | Sigma-Aldrich | Cat # L3755 |
| R848 (Resiquimod) | Invivogen | Cat # tlrl-r848-5 |
| CpG B (ODN 1826) | Invivogen | Cat # tlrl-1826 |
| Dulbecco's Phosphate-Buffered Saline (DPBS), (Corning®) | VWR | Cat # 45000-436 |
| 16% Paraformaldehyde (Formaldehyde) Aqueous solution, EM Grade | Thomas Scientific | Cat # 15710 |
| Taqman universal PCR master mix | Applied Biosystems | Cat # 4364338 |
| Bovine serum albumin standard grade | Gemini | Cat # 700-100P |
| UltraPure™ 1M Tris-HCl, pH 8.0 | Thermo Fisher Scientific | Cat # 15568025 |
| Tween-20 | Sigma-Aldrich | Cat # P1379-1L |
| LDS sample buffer 4x NuPAGE | Life Technologies | Cat # NP0007 |
| NuPAGE™ 10% Bis-Tris protein gels, 1.0mm, 12-well | Life Technologies | Cat # NP0302BOX |
| RPMI 1640, Corning® | VWR | Cat # 45000-396 |
| Gibco™ 2-Mercaptoethanol (55mM) | Fisher Scientific | Cat # 21985023 |
| EMD Millipore™ Poly-D-Lysine solution | Fisher Scientific | Cat # A003E |
| RPMI 1640 medium, powder (unbuffered) | Thermo Fisher Scientific | Cat # 31800022 |
| D-(+)-Glucose | Sigma-Aldrich | Cat # G8270-100G |
| DNase I from Bovine pancreas | Sigma-Aldrich | Cat # D4527 |
| Collagenase Type IV | Worthington Biochemical Corporation | Cat # LS004186 |
| Ammonium-Chloride-Potassium (ACK) lysis buffer | VWR | Cat # 118-156-101 |
| Fixable viability dye eFluor780 | eBioscience | Cat # 65-0865-14 |
| **Critical Commercial Assays** | | |
| Mouse IL-12(p70) ELISA Set | BD Biosciences | Cat # 555256 |
| Mouse IL-6 ELISA Set | BD Biosciences | Cat # 555240 |

| | | |
|---|---|---|
| Mouse IL-10 ELISA Set | BD Biosciences | Cat # 555252 |
| Mouse TNF (Mono/Mono) ELISA Set | BD Biosciences | Cat # 555268 |
| Mouse CXCL-10/IP-10/CRG-2 DuoSet ELISA kit | R&D Systems | Cat # DY466 |
| Quick-RNA Miniprep kit | Zymo Research | Cat # R1055 |
| High Capacity cDNA Reverse Transcription kit | Applied Biosystems | Cat # 4368814 |
| Griess reagent | Acros Organics | Cat # AC328670500 |
| Seahorse XF Cell Mito Stress Test Kit | Agilent | Cat # 103015-100 |
| **Experimental Models: Organisms/Strains** | | |
| C57BL/6 | Jackson Laboratory | Cat # 000664; RRID:IMSR_JAX000664 |
| C3HeB/FeJ | Jackson Laboratory | Cat # 000658; RRID:IMSR_JAX000658 |
| **Softwares and Algorithms** | | |
| FlowJo Software | TreeStar | N/A |
| Prism V 6.0 | Graphpad Prism | N/A |
| SoftMax Pro Software | Molecular Devices Corporation | N/A |
| **Other** | | |
| Fetal Bovine Serum (FBS) (A89C05C) | Gemini Bio Products | Cat # 100-106 |
| Penicillin/Streptomycin 100X | VWR | Cat # 45000-652 |
| L-Glutamine liquid | VWR | Cat # 45000-676 |
| Gentamycin | Thermo Fisher Scientific | Cat # 15750-060 |
| *cxcl10* Taqman Gene Expression Assay primer | Applied Biosystems | Mm00445235_m1 |
| *cyc (ppia)* Taqman Gene Expression Assay primer | Applied Biosystems | Mm02342430_g1 |
| *ifnb* Taqman Gene Expression Assay primer | Applied Biosystems | Mm00439546_s1 |
| *inos* Taqman Gene Expression Assay primer | Applied Biosystems | Mm00440502_m1 |
| *irf7* Taqman Gene Expression Assay primer | Applied Biosystems | Mm00516788_m1 |
| *isg15* Taqman Gene Expression Assay primer | Applied Biosystems | Mm01705338_s1 |
| *mx1* Taqman Gene Expression Assay primer | Applied Biosystems | Mm00487796_m1 |
| Immun-Blot low fluorescence PVDF membrane | Biorad | Cat # 1620264 |

## CONTACT FOR REAGENT AND RESOURCE SHARING

Further information and requests for resources and reagents should be directed to the Lead Contact Stefania Gallucci (gallucci@temple.edu).
